# Regulation of mitochondrial dysfunction induced cell apoptosis is a potential therapeutic strategy for herbal medicine to treat neurodegenerative diseases

**DOI:** 10.3389/fphar.2022.937289

**Published:** 2022-09-22

**Authors:** Ruo-Lan Li, Ling-Yu Wang, Hu-Xinyue Duan, Qing Zhang, Xiaohui Guo, Chunjie Wu, Wei Peng

**Affiliations:** ^1^ State Key Laboratory of Southwestern Chinese Medicine Resources, School of Pharmacy, Chengdu University of Traditional Chinese Medicine, Chengdu, China; ^2^ Hospital of Chengdu University of Traditional Chinese Medicine, Chengdu, China

**Keywords:** neurodegenerative disease, apoptosis, herbal medicine, mitochondrial dysfunction, therapeutic strategy

## Abstract

Neurodegenerative disease is a progressive neurodegeneration caused by genetic and environmental factors. Alzheimer’s disease (AD), Parkinson’s disease (PD), and Huntington’s disease (HD) are the three most common neurodegenerative diseases clinically. Unfortunately, the incidence of neurodegenerative diseases is increasing year by year. However, the current available drugs have poor efficacy and large side effects, which brings a great burden to the patients and the society. Increasing evidence suggests that occurrence and development of the neurodegenerative diseases is closely related to the mitochondrial dysfunction, which can affect mitochondrial biogenesis, mitochondrial dynamics, as well as mitochondrial mitophagy. Through the disruption of mitochondrial homeostasis, nerve cells undergo varying degrees of apoptosis. Interestingly, it has been shown in recent years that the natural agents derived from herbal medicines are beneficial for prevention/treatment of neurodegenerative diseases via regulation of mitochondrial dysfunction. Therefore, in this review, we will focus on the potential therapeutic agents from herbal medicines for treating neurodegenerative diseases via suppressing apoptosis through regulation of mitochondrial dysfunction, in order to provide a foundation for the development of more candidate drugs for neurodegenerative diseases from herbal medicine.

## 1 Introduction

Neurodegenerative diseases are characterized by slow and progressive dysfunction of neurons in certain areas of the brain and spinal cord (Ahat et al., 2019). Although the removal of redundant neuronal cells is essential for normal brain function, the abnormal death of different neuronal cell populations is a pathological sign of neurodegenerative diseases and will extend to other organs in the later stages of disease development (Gorman, 2008). The most common neurodegenerative diseases include a variety of complex dementia syndromes such as Alzheimer’s disease (AD), Parkinson’s disease (PD), Huntington’s disease (HD), frontotemporal dementia (FTD), mixed dementia (MD), and vascular dementia (VD). Besides, amyotrophic lateral sclerosis (ALS), and multiple sclerosis (MS) are also neurodegenerative diseases (Schrijvers et al., 2012). With the rapid growth of the elderly population worldwide, especially in developed countries, the prevalence of various age-related neurodegenerative diseases has increased sharply in recent years. It is worth noting that the incidence of AD, PD, and HD increases exponentially with age (Norton et al., 2014). According to the epidemical investigations, it is estimated that by 2050, the number of cases will increase sharply to 106.2 million. The huge medical expenses have an indelible impact on families and social economy (Tamburrino and Decressac, 2016). Therefore, exploring the pathogenesis of neurodegenerative diseases and discovering more novel effective treatments is an urgent need to alleviate the global upward trend of neurodegenerative diseases.

The researchers believe that genetic susceptibility and environmental factors, including paraquat, rotenone, metals such as iron, manganese and lead, and gaseous compounds such as carbon monoxide, contribute to the evolution of neurodegeneration (Liu et al., 2013). The etiology of neurodegenerative diseases is still in the exploratory stage. Taking AD as an example, many researchers believe that the apolipoprotein E4 gene can directly lead to excessive Aβ deposition in the brain of patients. The accumulation of aggregated protein is a common pathological feature of neurodegenerative diseases, which will lead to cell dysfunction. Besides these, the aggregated protein will cause cell death, leading to the occurrence and development of AD in turn (Baumgart et al., 2015; Livingston et al., 2017). However, after a large number of experimental verifications, it was found that the apolipoprotein E4 gene and related pathological features were not always found in AD patients, suggesting there are other causes of AD (Reitz and Mayeux, 2014). As a result, researchers put forward the mitochondrial cascade hypothesis. This hypothesis suggests that primary and secondary mitochondrial dysfunction is an important cause of AD, a driving force for Aβ plaque deposition in AD, and a typical event of neurodegeneration (Silva et al., 2011; Oliver and Reddy, 2019a).

In human body, the survival and activity of various cells are dependent on the production of energy. Mitochondria are the main production base of energy and are the key factors for generating transmembrane resting and action potentials (Johnson et al., 2021; Jessica et al., 2022). Energy can be produced through the Krebs cycle and the electron transport chain (ETC) in mitochondria (Lin and Beal, 2006). Nowadays, accumulating studies have indicated mitochondrial dysfunction and mitochondrial death may occur before the histopathological features of neurodegenerative diseases. Generally, mitochondrial DNA (mtDNA) in the human body will continue to produce new mutations as the body ages. When the mutations accumulate to a certain level, it will cause mitochondrial dysfunction, which in turn induces energy metabolism, oxidative stress and metabolic consequences (Naoi rt al., 2005; Armstrong, 2006; Schapira, 2006). The key consequences closely related to AD mainly include the destruction of intracellular calcium homeostasis and redox balance in neurons, activating the occurrence of intracellular apoptotic events, which ultimately promoting the development of neurodegenerative diseases (Swerdlow, 2018). At the same time, mitochondrial dysfunction can also interact with Aβ and p-tau to exacerbate the development of AD (Du and Yan, 2010; Spuch et al., 2012; Reddy and Oliver, 2019).

At present, the drugs used in neurodegenerative diseases cannot reverse the process of neuron loss, but only relieve the symptoms (Schneider et al., 2014; Sun et al., 2010; Solva et al., 2014). Consequently, it is of great significance to further understand the molecular mechanism behind it and discover more potential drugs for neurodegenerative diseases. For thousands of years, herbal medicine has accumulated a lot of experience in treating neurodegenerative diseases in practice. For example, in the *Huangdi Neijing* (*Huangdi’s Internal Classic*), it is recorded that single herbal medicine can be used to treat tremor, and modern pharmacological studies have further confirmed that a variety of active ingredients extracted from herbal medicine can be used to treat neurodegenerative diseases (Zhang et al., 2014; Hügel, 2015; Yang et al., 2017; Peng et al., 2022). Due to its wide curative effect and low side effects, herbal medicines have attracted increasing attention. Therefore, in this paper, we will focus on AD, PD and HD in neurodegenerative diseases, and introduce apoptosis related to mitochondrial dysfunction in neurodegenerative diseases and potential herbal medicines.

## 2 Mitochondrial homeostasis regulation

In the past, the classical doctrine considered mitochondria to be a static bean-like organelle. However, with the continuous development of electronic imaging technology, this doctrine has received a constant shock. In fact, as the most complex organelle in the cell, mitochondria are metabolically active and show high speed of movement in the cell. In other words, mitochondria are able to respond rapidly in various environments, which making them a dynamic organelle. Mitochondria can directly or indirectly regulate various signaling pathways in the organism, maintain calcium homeostasis, and synthesize energy for life activities mainly through the process of oxidative phosphorylation (OXPHOS) (Milane et al., 2015; Horbay and Bilyy, 2016). In neurons, mitochondria provide the energy needed to support neurite growth and neurotransmitter release, and the energy requirements are extremely high. However, the high energy production is accompanied by increased production of reactive oxygen species (ROS) in the cell (Mamelak, 2017). When intracellular mitochondrial homeostasis is dysregulated and the regulatory capacity of antioxidants is much lower than the rate of ROS production, ROS will accumulate excessively in the cell and induce a series of responses such as oxidative stress, which will eventually lead to cell death (Sarniak et al., 2016; Singh et al., 2019). Therefore, the regulation of mitochondrial homeostasis is crucial in the growth and development of neural cells.

### 2.1 Mitochondrial biogenesis

**FIGURE 1:**
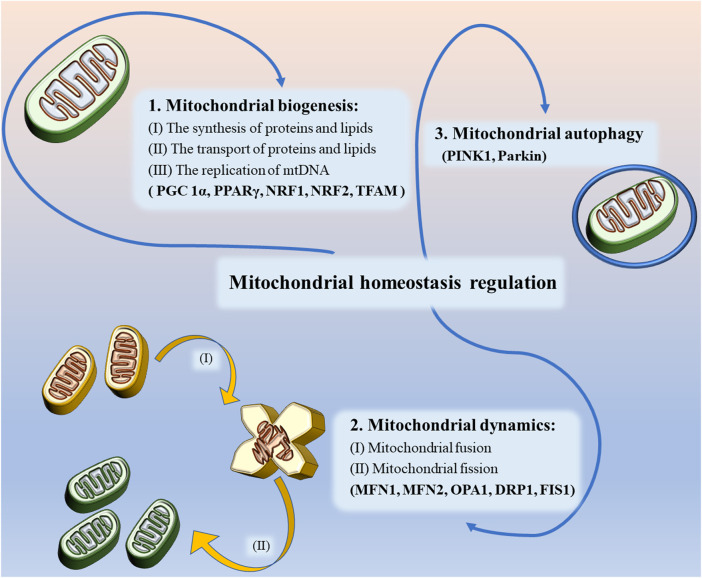
Mitochondrial homeostasis regulation includes regulation of mitochondrial biogenesis, mitochondrial dynamics and mitochondrial autophagy.

Mitochondrial biogenesis in neurons refers to the process of formation of new mitochondria, a process that involves the synthesis and transport of proteins and lipids, as well as the replication of mtDNA. The proteins required for mitochondrial biogenesis are mainly produced by ribosomes and later imported via outer membrane translocases. In the present study, most lipids were produced by endoplasmic reticulum and then transported to the mitochondria via vesicular transport or lipid transfer proteins, At the same time, there’s a small amount of lipid such as phosphatidylethanolamine, phosphatidylglycerol, and cardiolipin can also be generated directly in the mitochondria. Although mtDNA is the only type of DNA remaining in the cell other than nuclear DNA, it lacks a variable number of transfer RNA (tRNA) genes in its structure, so tRNA likewise needs to be transported from outside the mitochondria to inside the mitochondria (Wiedemann et al., 2004). As shown in Figure 1, mitochondrial biogenesis is mainly regulated by relevant transcriptional factors such as peroxisome proliferator-activated receptor γ (PPARγ) and coactivator 1α (PGC 1α) in the nucleus and mitochondria to increase the number of mitochondria by promoting protein synthesis and mtDNA replication, respectively (Manoli et al., 2007). In this process, nuclear respiratory factor 1 (NRF1) and nuclear respiratory factor 2 (NRF2) can be regulated by PGC 1α to bind to specific sites of mitochondrial transcription factor A (TFAM), thereby controlling the expression of multiple mtDNA-encoded genes, which contain oxidative phosphorylation system of mitochondria (OXPHOS) proteins and antioxidant-related proteins (Peng et al., 2017). In addition, AMP-activated protein kinase (AMPK) and silent mating-type information regulation 2 homolog 1 (SIRT1) regulate PGC 1α through phosphorylation and deacetylation when energy is scarce (Reznick and Shulman, 2006).

### 2.2 Mitochondrial dynamics

As mentioned earlier, mitochondria are dynamic organelles, and their movement is largely dependent on the strict regulation of mitochondrial dynamics to meet the energy and transport needs of the cell. Mitochondrial dynamics mainly includes fusion and fission (Valero, 2014; Peng et al., 2017). Mitochondrial fusion is a continuous process in which GTPases located on the outer mitochondrial membrane, namely outer membrane mitofusin 1 (MFN 1) and mitofusin 2 (MFN 2), tether the mitochondria to the endoplasmic reticulum and achieve mitochondrial fusion (Filadi et al., 2018). In contrast, the Optic Atrophy1 (OPA1) coordinates the mediation of mitochondrial inner membrane fusion and promotes the formation of a highly interconnected tubular network (Meyer et al., 2017). The formation of new mitochondria by fusion can repair damaged mitochondrial functions, provide conditions for efficient transfer of mtDNA during mitochondrial biogenesis and maintain cell survival, while also effectively preventing the accumulation of mtDNA mutations (Meeusen and Nunnari, 2005; Taguchi et al., 2007; Youle and van der, 2012; Burman et al., 2017).

However, in some pathological states, mitochondrial fusion is unable to repair damaged mitochondrial function, when mitochondrial fission is required (Flippo and Strack, 2017). During this process, the activity of dynamin-related protein 1 (DRP1), a key protein regulating fission and mitochondrial dynamic homeostasis, is controlled by several post-transcriptional modifications (PTM), the most important of which is the phosphorylation of residues (Ser616) and (Ser637) on DRP1 (Braschi et al., 2009; Zaja et al., 2014). Cyclin-dependent kinase 1 (CDK1) phosphorylates (Ser616) of Drp1, activating the mitochondrial fission activity of Drp1, while translocating to the outer mitochondrial membrane to interact with fission protein 1 (FIS1) and then forming a ring-like structure on the outer mitochondrial membrane to split the damaged mitochondria into two smaller spherical daughter mitochondria and eliminate the damaged portion (Smirnova et al., 2001; Zaja et al., 2014). However, in contrast, when the Ser637 site is phosphorylated by AMPK, the mitochondrial division activity will be inhibited (Li et al., 2015). During cell division, mitochondrial fission can ensure that dividing cells can obtain enough mitochondria to maintain normal cell life activities (Wai and Langer, 2015). In addition, the researchers found that downregulation of DRP1 expression could also indirectly enhance mitochondrial fusion (Wenger et al., 2013; Oliver and reddy, 2019b). Mitochondrial fusion and fission are self-balancing mechanisms necessary to maintain cellular activity, is fundamental to healthy metabolic function, and is an essential component in the regulation of mitochondrial homeostasis.

### 2.3 Mitochondrial autophagy

Mitochondrial autophagy refers to the removal of functionally impaired mitochondria from a cell through the mechanism of autophagy in order to maintain normal cellular activity. The process of catabolism, metabolism, and degradation that occurs in lysosomes is observed when paternal mitochondria are subject to degradation or when multiple diseases such as neurodegenerative diseases occur, and it is a form of quality control of healthy mitochondria (Lemasters, 2005). Studies have identified putative kinase 1 (PINK1) and Parkin as key molecules affecting mitochondrial autophagy. PINK1 is able to phosphorylate the Ser65 site of the ubiquitin-like structural domain, recruiting activation of the E3 ubiquitin ligase Parkin, which forms ubiquitin chains such as MFN1 and/or MFN2 with ubiquitin-binding proteins specific to mitochondrial autophagy on mitochondrial outer membrane proteins (Kondapalli et al., 2012; Lazarou et al., 2015; Wauer et al., 2015). MFN ubiquitination can promote mitochondrial autophagy and inhibit mitochondrial fusion (Ziviani et al., 2010; Scarffe et al., 2014). In addition, multiple mechanisms independent of PINK1 have been found in some studies to jointly maintain the initiation and regulation of mitochondrial autophagy in mitochondria when PINK1 is deficient (Liu et al., 2012; McWilliams et al., 2018).

## 3 Mitochondrial dysfunction and neuronal apoptosis

### 3.1 Mitochondrial dysfunction

Under physiological conditions, mitochondrial biogenesis, fusion, fission and autophagy work together to maintain mitochondrial homeostasis. Nevertheless, in some cases, mitochondrial dysfunction will lead to a series of consequences such as reduced ATP production and apoptosis (Kamil et al., 2021). In the present study, five possible mechanisms for mitochondrial dysfunction were identified, namely oxidative stress, mutation of mtDNA, opening of the mitochondrial permeability transition pore (mPTP), Ca^2+^ disruption, and reduced mitochondrial biosynthesis (Anna et al., 2020; Kang L. et al., 2020). Under physiological conditions, the intracellular ROS content is controlled within a certain range and the redox balance is achieved. However, when the balance between ROS and antioxidants is broken, mitochondrial dysfunction is caused by oxidative stress (Kung et al., 2021).

The mtDNA is a type of DNA that lacks protective histones and repair mechanisms and is highly susceptible to ROS attack. In this case, mtDNA is prone to mutation, causing damage to important functional regions within the genome such as the codon region of the oxidative phosphorylase gene and inhibit the expression and activity of OXPHOS proteins (Inna et al., 2009; Han and Chen, 2013). When mtDNA mutations accumulate to a certain threshold, mitochondrial dysfunction occurs. In addition, excessive ROS can reduce mitochondrial membrane potential and induce mPTP opening. When mPTP is irreversibly open for a long time, the mitochondrial inner membrane is fully depolarized, the inner membrane potential collapses, the OXPHOS process stops, and mitochondrial synthesis of ATP is terminated (Gjumrakch et al., 2011; Harper et al., 2004). At the same time, mitochondrial matrix efflux will also lead to massive superoxide anion production, membrane rupture, release of cytochrome C and apoptosis-inducing factors, etc. Ca^2+^ disruption is also an important factor contributing to mitochondrial dysfunction. It is known from previous studies that intracellular Ca^2+^ homeostasis can be regulated by mitochondria in conjunction with endoplasmic reticulum, etc. Ca^2+^ can enhance mitochondrial OXPHOS process and promote ATP synthesis, which in turn will affect calcium regulation. If the cell suffers from oxidative stress, mtDNA mutation or mTPT opening, it will likely cause the dysregulation of Ca^2+^ homeostasis in mitochondria, which will deepen the degree of mitochondrial dysfunction (Maassen et al., 2004; Sowers, 2004; Smith, 2007; Ito et al., 2021). From the above, it is clear that the various mechanisms complement each other and together contribute to the development of mitochondrial dysfunction. At the same time, ROS plays a central role in the pathogenesis of mitochondrial dysfunction, which runs through all the mechanisms of mitochondrial dysfunction.

### 3.2 Neuronal apoptosis

Apoptosis is a programmed form of cell death that is achieved by activating a specific program, the whole process is coordinated and smooth and can also be intervened, modified or stopped (Cotman and Anderson, 1995). In the initial phase of apoptosis, we can clearly observe cell shrinks, morphological rounding and loss of contact with surrounding cells. This is followed by dilation of the endoplasmic reticulum in the cytoplasm and swelling of the cisternae to form vacuoles and vesicles. In addition, nuclear modifications were taking place in parallel with it. Chromatin in the nucleus coalesces into a compact mass, which is internucleosomal disjointed by endonucleases (Tang et al., 2019; Sharma et al., 2021a). Apoptosis can affect different cell populations, which include neural precursor cells and differentiated neurons and glial cells, among others. Apoptosis ensures that only morphologically and functionally normal cells survive and are properly connected to their axons and synapses. What’s more, it is essential for nerve function. Too much or too little can lead to a variety of pathological states (Sharma et al., 2021b).

**FIGURE 2:**
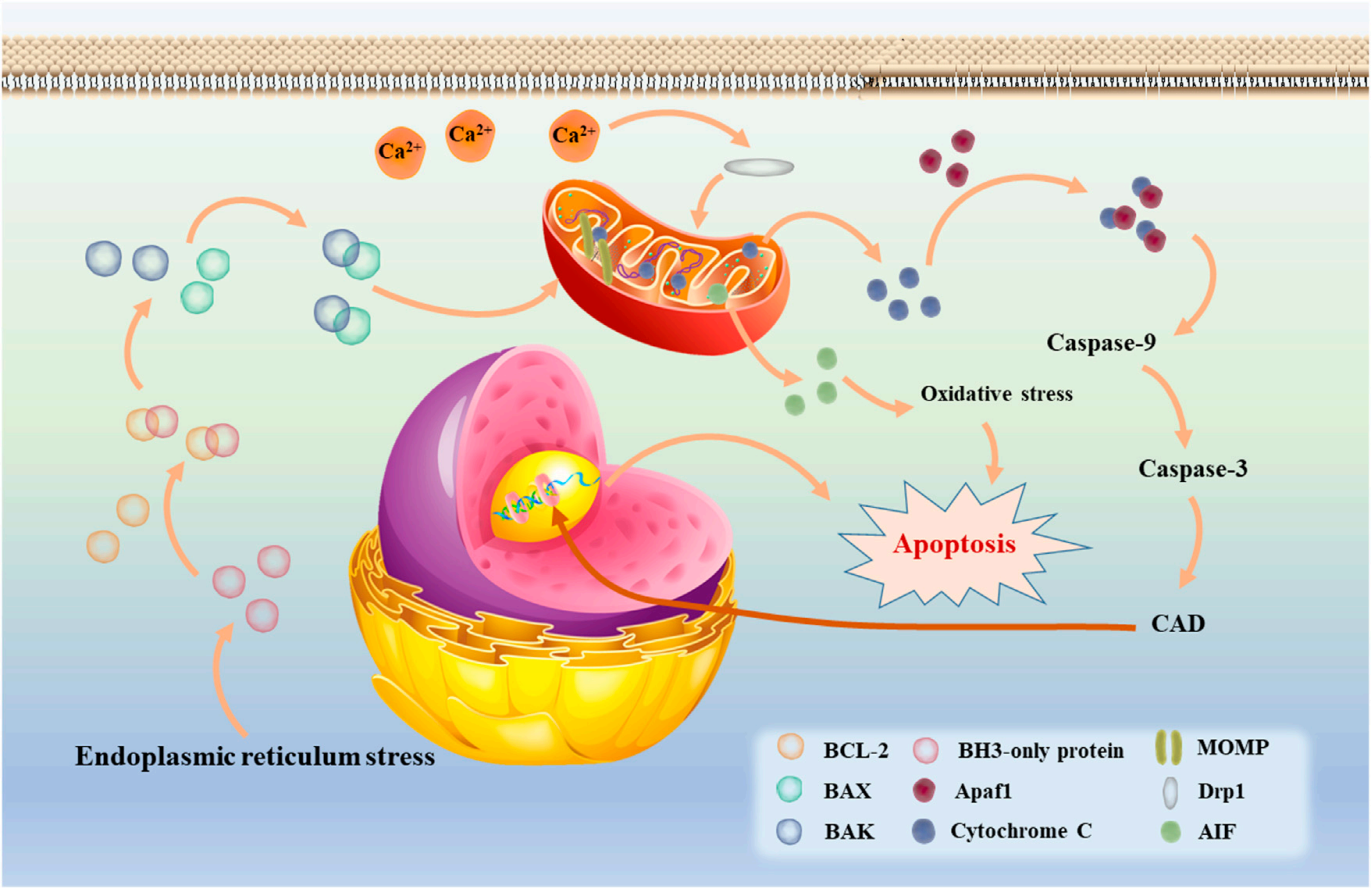
The intrinsic apoptotic mediated by mitochondria (also called Bcl-2 or mitochondrial pathway).

Apoptosis can be triggered by a number of pathways, which are nowadays divided into two main categories, namely the extrinsic pathway (also known as the death receptor pathway) and the intrinsic pathway. Although the two theories involve different pathways, both ultimately form apoptotic vesicles that are recognized and cleared by phagocytes (Satija et al., 2021). In the extrinsic pathway, FAS, a member of the tumor necrosis factor (TNF) superfamily, can cleave and activate caspase-8 by binding to FAS ligands to form the intracellular death-inducing signaling complex (DISC), which in turn activates the downstream caspases (caspase-3 and -7) to achieve cell death (Felderhof-Mueser et al., 2002). During this process, the extrinsic pathway of apoptosis is linked to the intrinsic pathway due to the involvement of caspase-3. The intrinsic apoptotic pathway, also known as the Bcl-2 or mitochondrial pathway, is where cytochrome C plays an extremely important role. As shown in Figure 2, when intracellular stress occurs (e.g., growth factor deprivation, endoplasmic reticulum stress, etc.), the amount of free BCL-2 decreases due to increased production of BH3-only protein, a key initiator of apoptosis, which increases the binding of the anti-apoptotic protein BCL-2 to it (Puthalakath and Strasser, 2002; Chen et al., 2005). It is reported that activation of the pro-apoptotic proteins BAX and BAK results in the formation of oligomers that induce the mitochondrial outer membrane permeablisation (MOMP) and the release of cytochrome C (Huang and Strasser, 2000; Kelly and Strasser, 2020). After release into the cytoplasm, cytochrome C activates caspase-3 and -9 via forming an apoptosome complex by binding to apoptotic protease factor 1 (Apaf 1) (Yuan et al., 2013). It has been shown experimentally that activation of caspase-3 leads to a significant release of caspase-activated deoxyribonuclease (CAD), resulting in DNA fragmentation in the cell and apoptotic phenomena such as chromatin shrinkage (Enari et al., 1998; Shakeri et al., 2017; Nijhawan et al., 1997). It is well established that when Ca^2+^ signaling between the endoplasmic reticulum and mitochondria is disrupted, BCL-2 family proteins will also be activated. Subsequently, the localization of Drp1 to mitochondria is further enhanced, which greatly increase mitochondrial division and the amount of mitochondrial fragmentation, resulting in mitochondrial dysfunction, which not only releases cytochrome C, but also releases apoptosis-inducing factor (AIF) to increase oxidative stress and induces apoptosis (Jeong and Seol, 2008). In addition, it is known that cardiolipin is a lipid synthesized in mitochondria and is specific to mitochondria. When oxidation of cardiolipin occurs, it can also cause MOMP and promote the release of cytochrome C (Ott et al., 2002; Gonzalvez and Gottlieb, 2007; Dingeldein et al., 2018). Therefore, mitochondrial dysfunction plays a key role in the endogenous pathway of apoptosis.

## 4 Neuronal degenerative diseases and herbal medicine

In neurodegenerative diseases, apoptosis is an important way that mediates neuronal cell death and it is essential for cell survival and homeostasis regulation. However, when apoptosis is abnormally increased, it may lead to neurodegenerative diseases. At the same time, the intrinsic apoptotic pathway is an important way of neurodegenerative diseases. Compared with other diseases, the development of therapeutic drugs for neurodegenerative diseases is relatively slow. Surprisingly, herbal medicine has many effects on endogenous apoptosis in neurodegenerative diseases, among which mitochondrial dysfunction plays an important role.

### 4.1 Alzheimer’s disease

#### 4.1.1 Mitochondrial dysfunction and AD in neuronal apoptosis

Alzheimer’s disease (AD) is a typical neurodegenerative disease, accounting for 70%–80% of dementia. The incidence of AD is increasing with age. What’s more, there is an increasing prevalence of the disease among relatively young groups. Up to now, the number of AD patients in the world is up to 50 million. It is estimated that the number will double by 2050 (Bhute et al., 2020). Existing drugs have poor efficacy and cannot cure or reverse the pathogenesis of AD, which brings great economic burden to patients and the society. Existing studies have found that malnutrition, lack of physical exercise, infection and other factors may lead to AD, while depression, hyperlipidemia, cardiovascular and cerebrovascular diseases, diabetes and other complications can multiply the risk of AD (Sharma and Singh, 2020b). Currently, researchers have carried out extensive and in-depth exploration on the pathogenesis of AD, and put forward a variety of theories, including cholinergic theory, oxidative stress theory, Aβ cascade reaction and inflammation hypothesis (Su et al., 1994). But what is frustrating is that researchers still failed to accurately explain its underlying mechanism. The clinical symptoms of AD may be characterized by progressive cognitive decline, accompanied by senile plaques composed of β-amyloid peptide (Aβ) and neurofibrillary tangles composed of hyperphosphorylated tau (Anandatheerthavarada et al., 2003). Aβ has strong neurocytotoxicity and can activate intracellular apoptotic pathways through a variety of pathways, thereby inducing apoptosis and leading to pathological changes and dementia ultimately (Zhan et al., 2018; Nishizaki, 2019). It is generally believed that the deposition of Aβ and excessive ROS are involved in many pathways of endogenous apoptosis, while multiple signaling pathways related to exogenous apoptosis, such as TLR4/NF-KB and MAPKs, can also be involved in the endogenous apoptosis. For example, Aβ and ROS can initiate the phosphorylation of JNK by activating TLR4, and the phosphorylated JNK can migrate to the nucleus through c-Jun and AP-1 trans-activation to transcribed apoptosis-related genes and promote the activation of Bad and Bax. In fact, many studies have shown that amyloid precursor protein (APP) can be transported to the mitochondrial membrane and cleaved by γ secretase to form Aβ. In mitochondria, Aβ can destroy mitochondrial membrane potential by interacting with a variety of proteins, causing mitochondrial dysfunction and releasing a large amount of cytochrome C to induce apoptosis (Lustbader et al., 2004; Du et al., 2008).

**FIGURE 3:**
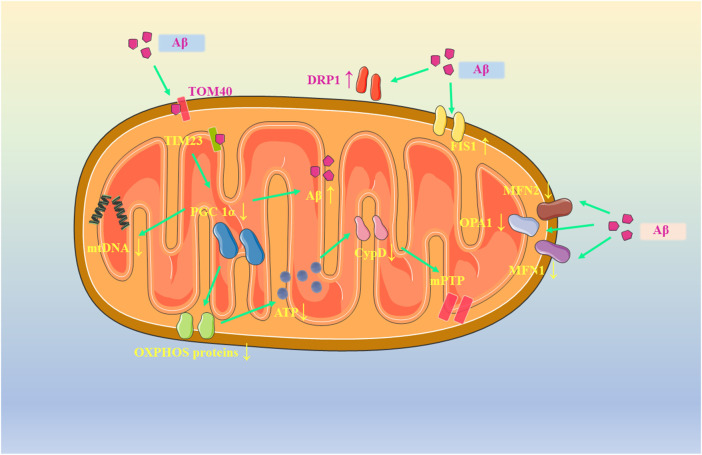
Mitochondrial dysfunction in AD.

As shown in Figure 3, in the current studies, Aβ was found to disrupt mitochondrial function and induce apoptosis by affecting various processes such as mitochondrial biogenesis, mitochondrial metabolism, division, fusion, and autophagy. First, Aβ can enter into the mitochondria by interaction with TOMM40 and TIM23 and then cause oxidative damage to mtDNA in the mitochondrial biogenesis phase. Transfection of AD-associated mtDNA produced hybrids with increased levels of fragmented mtDNA and a direct 50% decrease in mtDNA transcription, promoting the onset of mitochondrial dysfunction while showing the characteristics such as respiratory enzyme defects and increased ROS production (Reddy et al., 2018; Maiti et al., 2019). Subsequently, reduced expression of PGC1α, which is closely associated with biogenesis, was found *in vivo* and *in vitro* as well as brain tissue of patients. It was experimentally verified that PGC1α overexpression prevented nitrosative stress, reversed mitochondrial dysfunction and reduced neuronal apoptosis by enhancing OXPHOS levels. In neurons transfected with AD-related genes or after Aβ treatment, OXPHOS-related protein expression was reduced, with the resultant decrease in mitochondrial membrane potential, reduced mitochondrial respiration rate and reduced ATP production (Kuruva et al., 2017; Reddy et al., 2018; Maiti et al., 2019). In addition, the reduction in ATP would also decrease the expression of cyclophilin D (Cyp D), a mitochondrial matrix protein, causing a prolonged opening of the mitochondrial permeability transition pore (PTP) and ultimately apoptosis (Reddy et al., 2016). Surprisingly, PGC1α not only affects mitochondrial biogenesis, but also inhibits Aβ production by reducing the amount of β-APP cleaving enzyme (BACE1) and Aβdegrading enzymes neprilysin (Katsouri et al., 2011). In addition, its downstream target Nrf2 can also promote autophagy through the p62 pathway, leading to a decrease in Aβ (Joshi et al., 2015).

During the mitochondrial dynamics, Aβ has been shown to impair neuronal mitochondrial motility without destabilizing microtubules, causing an imbalance in mitochondrial dynamics and presenting a state of low-fusion and high-fission. In transgenic and Aβ peptide injected rat models, increased Aβ directly resulted in increased expression of mitochondrial fission proteins DRP1 and FIS1 and increased GTPase activity in neurons, whereas expression of fusion proteins MFN1, MFN2 and OPA1 was directly reduced (Wang L. et al., 2016; Kandimalla et al., 2016; Xu L. L. et al., 2017; Kandimalla et al., 2018). In contrast, when DRP1 expression is suppressed, β-secretase 1 (BACE1) expression in the brain will decrease and cognitive function is improved.

#### 4.1.2 Potential therapeutic agents from herbal medicine for mitochondrial dysfunction in AD

*Panax ginseng* has been used as a tonic medicine in China for thousands of years. However, in recent studies, several active components contained in ginseng were found to have ameliorative effects on mitochondrial dysfunction in AD. Ginsenoside Rb1 inhibited mitochondrial dysfunction by reducing Bax and Cleaved Caspase-3 levels and upregulating Bcl-2 levels in the hippocampus with Aβ_1-40_ induced AD rats, thereby inhibiting neuronal apoptosis and improving learning and memory abilities of rats in spatial navigation (Wang et al., 2018). In 3xTg-AD mice, ginsenoside Rg1 upregulated complexxin-2 (CPLX2) and synaptosomal-associated protein 25 (SNP25), restoring mitochondria-related functions and improving behavioral deficits in mice (Nie et al., 2017). In a rat AD model induced by d-gal, ginsenoside Rg3 inhibited apoptosis by upregulating Bcl-2 expression and inhibiting caspase-3, caspase-9, Bax and AIF expression. Also, Rg3 could inhibit the expression of Cyt C in cells, suggesting that Rg3 may inhibit apoptosis through the mitochondrial pathway (Lee et al., 2013). Ginsenoside Rd, another important saponin-like component of ginseng, inhibited apoptosis induced by Aβ_25-35_ in hippocampal neurons by upregulating mRNA of Bcl-2 and downregulating mRNA of Bax and Cyt C (Liu et al., 2015). The same protective effect was found in the study of ginsenoside Re. In Aβ_25-35_-induced SH-SY5Y cells, ginsenoside Re increased the expression of Nrf2 and the ratio of Bcl-2/Bax, and inhibited the release of Cyt C and the protein levels of caspase-3 and caspase-9 in mitochondria (Liu et al., 2019). In Aβ-injected mice, ginsenoside Re also reversed learning and memory deficits in mice by restoring amino acid, lecithin and sphingolipid metabolism (Li et al., 2018). Thus, both *in vitro* and *in vivo* experiments suggest a possible protective effect of ginsenoside Re against AD. In addition to the above ginsenoside active monomer components, red ginseng oil (RGO) also exerted protective effects on Aβ_25-35_-induced PC12 cells. RGO could reduce BACE1 activity, inhibit apoptosis-related molecules such as Bax, caspase-3 and PARP-1, and protect neural cells from apoptotic damage. Meanwhile, it was found that RGO could inhibit the inward flow of Ca^2+^ in mitochondria, reverse the mitochondrial membrane potential, and reduce the content of ROS in cells, suggesting that RGO could protect neural cells by protecting the normal function of mitochondria and inhibiting apoptosis (Lee et al., 2017). Korean red ginseng extract (KRG) has been studied as a multi-component drug for AD in the clinical phase II. *In vitro* experiments, KRG inhibited apoptosis by activating the PI3K/Akt signaling pathway, suppressed NF-κB expression and decreased mitochondrial membrane potential, and also restored mitochondrial respiration associated with ATP production (Nguyen et al., 2015; Shin et al., 2019; Choi et al., 2020). In addition, in 5XFAD mice that expressed five familial AD mutations, it was further demonstrated that KRG could inhibit the occurrence of mitochondrial dysfunction by protecting the mitochondrial fusion and fission processes, thereby protecting AD mice (Shin et al., 2019). In conclusion, multiple active components of ginseng can play a protective role on mitochondrial dysfunction in AD. Unfortunately, it has not been specifically elucidated in some of the experiments what activities of the active ingredients influenced to restore mitochondrial function, and this needs to be further elucidated in future experiments.

In addition to ginseng, active monomeric components or extracts of many herbs can inhibit apoptosis by reversing mitochondrial dysfunction in AD. For example, puerarin from *Pueraria lobata* can reverse the increased expression of Bax and the decreased expression of Bcl-2 and p-BAD in Aβ_25–35_-induced PC12 cells through PI3K signaling pathway, and also inhibit the release of cytochrome C, which ultimately reducing cell apoptosis (Xing et al., 2011). Subsequently, researchers investigated the protective effects of puerarin on cells using mitochondrial transgenic neuronal cell cybrid models of sporadic AD. The results revealed that puerarin inhibited the generation of endogenous ROS in cells and suppressed the activation of caspase-3, p38 and JNK (Zhang et al., 2011). Urolithin A (UA), mainly produced by the metabolism of ellagitannins in pomegranate fruits and walnuts in the intestine, has the potential to penetrate BBB (Cásedas et al., 2020). Previous studies have found that UA could inhibit oxidative stress and mitochondrial autophagy in cells, thus playing a neuroprotective role (González-Sarrías et al., 2017; Pradeepkiran et al., 2022). One step further, in the latest study, it was found that UA could increase expression of genes for mitochondrial biogenesis and OXPHOS in SH-SY5Y cells transfected with the amyloid-precursor protein 695 (SY5Y-APP695) (Esselun et al., 2021). Other potential therapeutic agents from herbs are shown in Supplementary Table S1.

### 4.2 Parkinson’s disease

#### 4.2.1 Mitochondrial dysfunction and AD in neuronal apoptosis in PD

Due to the global demographic transition, Parkinson’s disease (PD) has become the second most common neurodegenerative disease after AD (de Lau and Breteler, 2006). Clinical symptoms of PD can manifest as non-motor symptoms such as insomnia and dementia, as well as motor dysfunction, including bradykinesia, resting tremor and muscle tonus (Tysnes and Storstein, 2017). In China, PD affect about 3.9% of older people aging 50 years old, and this number will grow to 4.94 million by 2030, narrating half of the people with PD worldwide (Dorsey et al., 2007). PD can be induced by a combination of genetic factors (familial PD) and environmental factors (Sporadic PD) (Koros et al., 2017). The pathological features of PD are mainly manifest as retarded dopaminergic neurons in region of substantia nigra pars compacta and the aggregation of Lewy vesicles containing misfolded α-synuclein (α-syn). (Dickson, 2018). Although the genetic and cellular processes of PD have been extensively studied in recent decades, the exact pathogenesis of the disease has not been well explained. There is growing evidence that mitochondrial dysfunction-induced apoptosis is closely related to neurological damage in PD.

**FIGURE 4:**
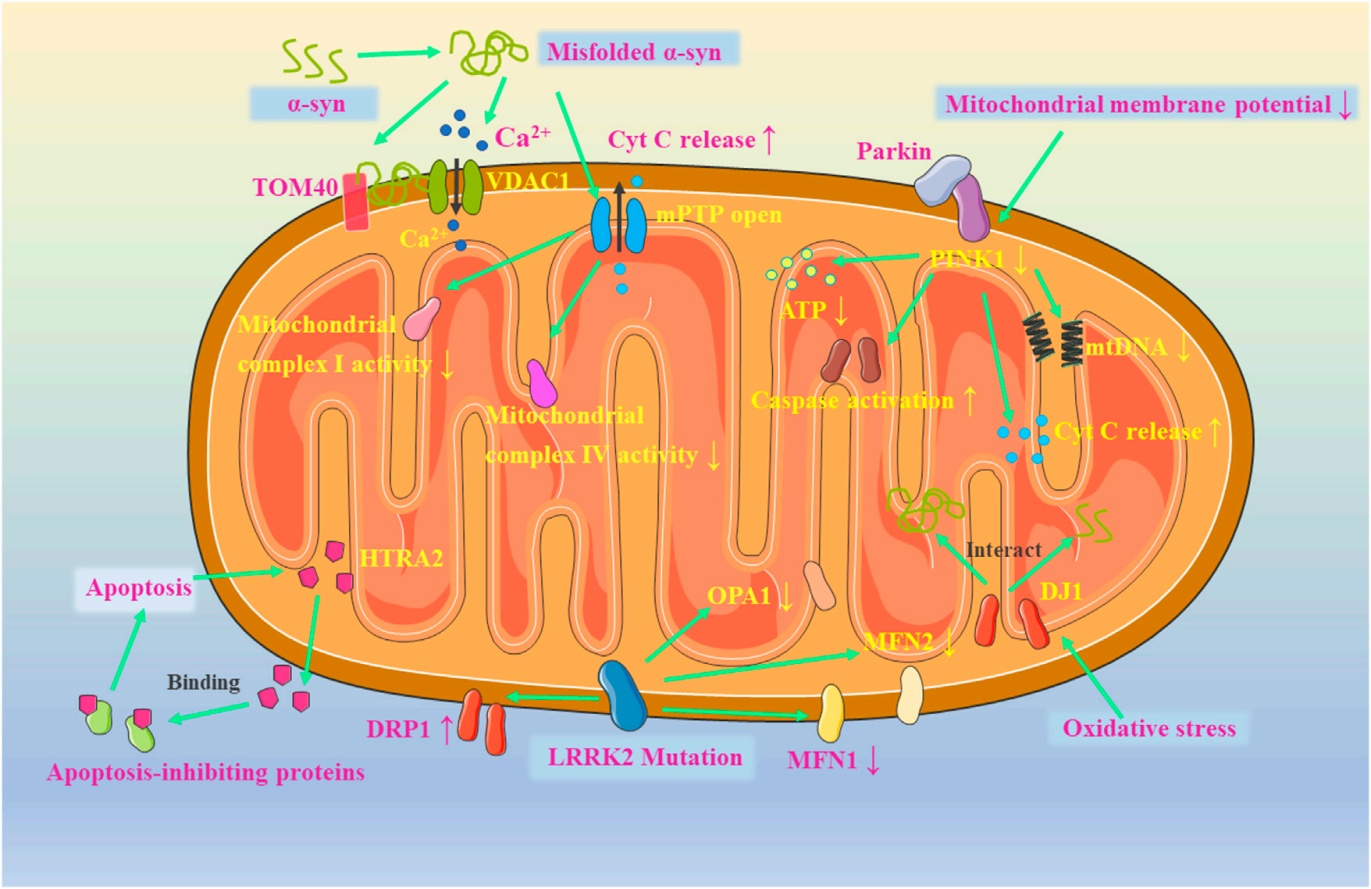
Mitochondrial dysfunction in PD.

Genetic and environmental factors can affect mitochondrial function through a variety of pathways, including those affecting mitochondrial biogenesis, mitochondrial dynamics, mitochondrial autophagy, aberrant electron transport chain complexes, Ca^2+^ homeostasis, axonal transport, and reactive oxygen species production (Georgiou et al., 2017; Billingsley et al., 2019; Sun et al., 2019). All these functional changes will cause mitochondrial dysfunction and neuronal apoptosis, which in turn induce PD. α-synuclein plays a critical role in dopamine synthesis, storage, and release in dopaminergic neurons, while it also can be imported into mitochondria membrane (Burré, 2015). Under physiological conditions, misfolded α-syn can be cleared by the ubiquitin-proteasome and autophagy-lysosomal systems. However, when the generation of misfolded α-syn exceeds the cellular clearance capacity, α-syn aggregates to form oligomers and generates insoluble protofibrils which are the core of Lewy vesicles, and thus lead to the development of neurotoxicity (Parnetti et al., 2019). In new studies it has been shown that α-syn interacts with outer mitochondrial membrane proteins such as translocase of the outer membrane 20 (TOM20) and voltage-dependent anion-selective channel 1 (VDAC1), cutting off transport channels for proteins required for mitochondrial biosynthesis and inhibiting mitochondrial-endoplasmic reticulum interactions to dysregulate intracellular Ca^2+^ homeostasis, which are shown in Figure 4 (Guardia-Laguarta et al., 2014; Di Maio et al., 2016; Paillusson et al., 2017). In addition, aggregated or mutated α-syn can disrupt mitochondrial kinetic homeostasis and autophagic processes by regulating the actin cytoskeleton and inducing the opening of the mitochondrial permeability transition pore (PTP) (Ludtmann et al., 2018). After mitochondrial dysfunction, mitochondrial complex I and IV activities are inhibited, oxidative stress is enhanced, cytochrome C is released, and apoptosis occurs in neuronal cells (Paillusson et al., 2017). At the same time, a mitochondrial matrix protease, ATP-dependent Clp protease (ClpP), was discovered in recent studies, which can reduce the formation of α-synuclein fibrillary by inhibiting the phosphorylation of α-synuclein Ser129. This finding suggests that ClpP may be an effective therapeutic target for α-synuclein induced PD (Hu and Wang, 2019).

In addition to α-syn, genes such as Parkin, PTEN-inducible putative kinase 1 (PINK1), leucine-rich repeat kinase 2 (LRRK2) and DJ-1 have also shown a high correlation with mitochondria in PD. The role of the PINK1/Parkin pathway in the maintenance of mitochondrial quality control has been thoroughly investigated, while its role in PD pathology have also been progressively identified. PINK1 can recruit Parkin into dysfunctional mitochondria and regulate mitochondrial dynamics by activating mitochondrial fission or mitochondrial autophagy (Gegg et al., 2009; Pickles et al., 2018). Also, PINK1 can inhibit apoptosis by suppressing cytochrome C release and caspase activation (Petit et al., 2005). However, when the mitochondrial membrane potential is impaired on a large scale, the stability of PINK1 at the outer mitochondrial membrane is affected by the ripple effect. PINK1 deficiency leads to altered mitochondrial morphology and impaired autophagy, while evidence suggests that this defect also impairs mtDNA and ATP levels during mitochondrial biogenesis (Tufi et al., 2014). In addition, PINK1, if overexpressed in PD models, can protect neuronal cells by inducing α-syn autophagy (Liu J. et al., 2017). Mutations in LRRK2 are the most common cause of autosomal dominant familial PD and some sporadic PD. LRRK2 can mediate mitochondria by co-localizing with the kinetic superfamily of GTPases (Drp1, Mfn1, Mfn2 and OPA1) Kinetics (Stafa et al., 2014). Oral administration of low doses of rotenone results in mice with LRRK2 mutations exhibiting significant motor defects. Mutations in LRRK2 will result in increased DRP1 Ser616 phosphorylation and mitochondrial fission activity, and mitochondrial dysfunction will occur (Liu H. F. et al., 2017). Mutations in the DJ1 gene are associated with autosomal recessive juvenile PD. When cells undergo oxidative stress, DJ1 cysteine residues (C106) acidify and localize to mitochondria, interacting with monomeric or multimeric α-syn to inhibit oligomer formation thereby exerting a protective effect (Canet-Avilés et al., 2004). In addition, when mitochondria are stimulated by apoptosis, high-temperature requirement protein A2 (HTRA2) can be released from the mitochondrial membrane gap and bind to apoptosis-inhibiting proteins to further activate caspase activity and caspase-independent death (Martins et al., 2004). In contrast, under normal physiological state, HTRA2 can be responsible for the degradation of denatured proteins in mitochondria and can exert neuroprotective effects by targeting DJ1 mutations (Dagda and Chu, 2009).

#### 4.2.2 Potential therapeutic agents from herbal medicine for mitochondrial dysfunction in PD

As mentioned above, PD is closely related to mitochondrial dysfunction, so mitochondrial dysfunction can be found in many studies on herbal therapy for PD. Theacrine is one of the main purine alkaloids in tea. It is easily absorbed by the gastrointestinal tract and can penetrate the central nervous system. Therefore, in recent years, attempts have been made to apply it to neurodegenerative diseases such as PD, with surprising results. In 6-hydroxydopamine (6-OHDA)-treated rats and MPTP-treated mice/zebrafish, theacrine prevented the loss of dopaminergic neurons and impaired behavioral performance. What’s more, 1-methyl-4-phenylpyridinium (MPP+)-treated SH-SY5Y cells showed that theacrine could prevent cell apoptosis through SIRT3-mediated SOD2 deacetylation, reduce ROS accumulation and restore mitochondrial function, thus alleviating cell apoptosis caused by oxidative damage and mitochondrial dysfunction (Duan et al., 2020). Unfortunately, it has not been clarified in this study what biological functions of mitochondria are mediated by theacrine.

Quercetin is a common flavonoid with antioxidant activity, which plays a neuroprotective role in a variety of neurodegenerative diseases. Notably, in (MPP+) -induced SH-SY5Y cells, quercetin preconditioning effectively protected cells from mitochondrial damage in a dose-dependent manner. For example, quercetin could protect mtDNA replication and transcription in mitochondria by upregulating TFAM expression, and could also up-regulate the expression of H2AX and dopaminergic neuron marker tyrosine hydroxylase (TH). However, it has no significant effect on the expression of ND9, a nuclear coding subunit of complex I in plasmid electron transport chain, and PGC-1α. Through above measures, quercetin can effectively reverse mitochondrial dysfunction induced by MPP+, and ensure the normal production of ATP and the balance of ROS in cells (Kang S. et al., 2020).

Baicalein is one of the main flavonoids in herbal medicine *Scutellaria baicalensis*, which can be used to treat inflammation, hypertension and cardiovascular diseases in modern pharmacological studies. After years of research, it has been found that baicalein also has significant efficacy on PD, and the clinical trial phase I has been completed in China. Has neuroprotective effect in various PD models, and its protective effect is directly related to mitochondria mediated apoptosis. For example, in 6-OHDA-induced SH-SY5Y cells, baicalin could reverse MMP, inhibit the activation of mitochondrial downstream caspase-9 and caspase-3, and reduce the protein level of phosphorylated JNK (Lee et al., 2005). In rotenone-induced PC12 cells, baicalein could inhibit ROS production, MMP dissipation and ATP deficiency. Meanwhile, the activation of caspase-3 and caspase-7 was down-regulated by baicalein (Li et al., 2012). The similar results were found in isolated rat brain mitochondria, while the protection effect of baicalein was performed by promoting active mitochondrial respiration and inhibiting mitochondrial swelling (Li et al., 2012). In another study, N2A cells were co-transfected with E46K α-synuclein to establish PD models, and it was found that baicalein could reduce mitochondrial depolarization (Jiang et al., 2010). In the *in vivo* model, intraperitoneal injection of baicalein for MPP + induced rats could reduce α-synuclein aggregates and inflammation-related factors in rats. In addition, baicalein could also inhibit the activation of caspase-9 and caspase-12 in the dopaminergic system of rat substantia nigra striatum (Hung et al., 2016). Other potential therapeutic agents from herbal medicine for mitochondrial dysfunction in PD are shown in Table 1.

**TABLE 1:** Mitochondrial dysfunction-associated natural products for PD.

Extracts/monomers	Source	*In vitro* mode	*In vivo* model	Mechanisms or effects	Clinical trial	Chemical structure	Refs
Theacrine (100 and 200 μM)	*Camellia sinensis* (L.) O. Kuntze	MPP-treated SH-SY5Y cells	—	Activates SIRT3 to deacetylate SOD2 and restore mitochondrial functions; preventing apoptosis	—	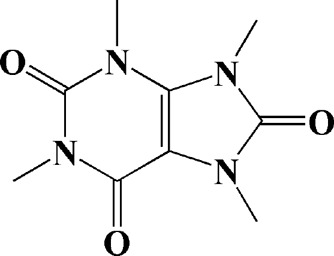	**Duan et al. (2020)**
(10, 20 mg/kg/d)		—	6-OHDA-treated rats, MPTP-treated mice/zebrafish		—		**Duan et al. (2020)**
Quercetin (1 μg/ml)		1-methyl-4-phenylpyridinium (MPP+) -induced SH-SY5Y cells	—	Increasing expression levels of tyrosine hydroxylase and mitochondria controlling proteins TFAM, H2AX and tyrosine hydroxylase (TH), a marker for dopaminergic neuron; alleviating the fragmentation of mitochondria	—	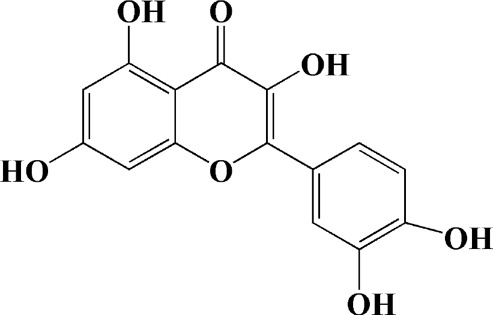	**Kang et al. (2020b)**
Baicalein (12.5 μM)	Scutellaria baicalensis	6-OHDA-induced SH-SY5Y cells	—	Protecting MMP and caspase cascades downstream of mitochondria; inhibiting caspase-9 and caspase-3 activation; inducing a significant reduction in the level of phospho-JNK	Phase I	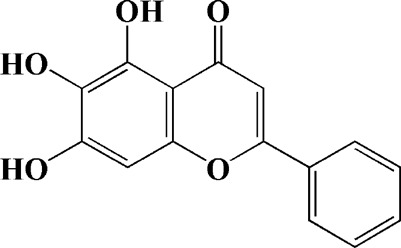	**Lee et al. (2005)**
(40 μM)		Rotenone-induced PC12 cells	—	Suppressing apoptosis; inhibiting the accumulation of ROS, ATP deficiency, mitochondrial membrane potential dissipation, and caspase-3/7 activation			**Li et al. (2012)**
(0.5, 5μM)		Isolated rat brain mitochondria	—	Preventing ROS production, ATP deficiency and mitochondrial swelling			**Li et al. (2012)**
(10 μM)		N2A cells co-transfected with E46K α-syn construct and pTK-Hyg plasmid	—	Attenuating mitochondrial depolarization and proteasome inhibition; decreasing formation of E46K α-syn-induced aggregates			**Jiang et al. (2010)**
(30 mg/kg/d)		—	MPP + -induced SD rats	Suppressing α-synuclein aggregates, ED-1, activated caspase-1, IL-1β and cathepsin B; inhibiting activation of caspases 9 and 12, as well as LC3-II levels			**Hung et al., (2016)**
Astragaloside IV (50 μM)	*Astragalus* membraneaceus Bunge	(LPS/MPP+)-induced astrocyte	—	Increasing expression of PINK1 and Parkin; decreasing the level of TOM20; reducing mitochondrial ROS generation	—	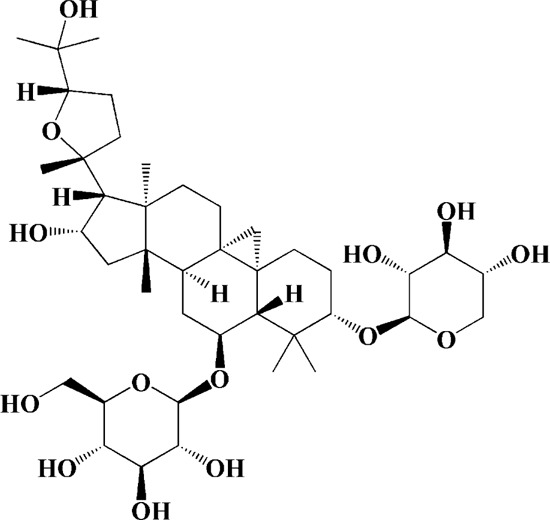	**Xia et al., (2020)**
Extract of *Humulus japonicus* (50, 100 and 200 μg/ml)	*Humulus japonicus*	6-OHDA-induced SH-SY5Y cells	—	Decreasing expression of cleaved caspase-9, cleaved caspase-3 and cleaved PARP; suppressing Cytochrome c release; inhibiting phosphorylation of ERK1/2	—	—	**Ryu et al., (2017)**
Gastrodin (1, 5, 25 μM)	Gastrodia elata	(MPP+) -induced SH-SY5Y cells	—	Activating p38 MAPK/Nrf2 signaling pathway; increasing Nrf2 nuclear translocation and HO-1 expression; increasing membrane potential and Bcl-2/Bax ratio	—	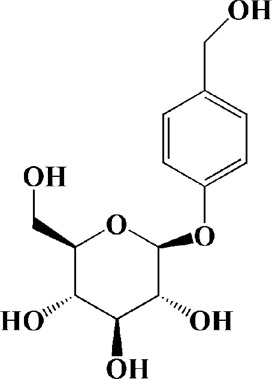	**Jiang et al., (2014)**
Extract of *Gastrodia elata* Blume (10, 100, 200 μg/ml)	*Gastrodia elata* Blume	(MPP+)-treated MN9D dopaminergic cells	—	Attenuating the elevation of ROS; decreasing Bax/Bcl-2 ratio and poly (ADP-ribose) polymerase proteolysis	—	—	**Kim et al., (2011)**
2-[4-hydroxy-3-(4-hydroxybenzyl)benzyl]-4- (4-hydroxybenzyl)phenol (0.01, 1 μM)		Rotenone-induced PC12 cells	—	Activating Nrf2/ARE/HO-1 signaling pathway; increasing MMP; reducing ROS accumulation	—	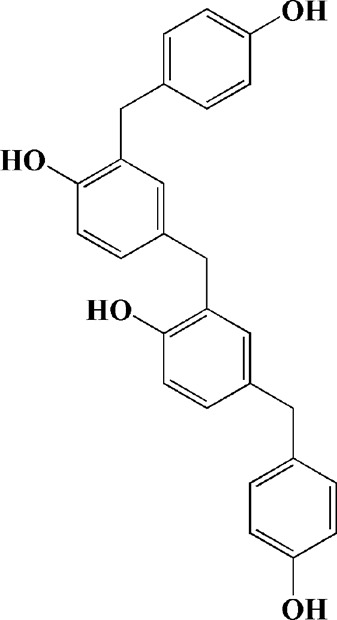	**Huang et al., (2016)**
Extract of Cuscutae Semen (0.01, 5 and 10 μg/ml)	Cuscutae Semen	(MPP+) -induced PC12 cells	—	Inhibiting ROS generation; suppressing glutathione peroxidase activation	—	—	**Ye et al., (2014)**
Extract of *Rhus verniciflua* Stokes (10 μg/ml)	*Rhus verniciflua* Stokes	Rotenone-induced SH-SY5Y	—	Attenuating the up-regulation of Bax, Caspase-9 and Caspase-3 and down-regulation of Bcl-2; inhibiting MMP disruption	—	—	**Sapkota et al., (2011)**
Panaxatriol saponins (0.01, 0.03, 0.06 and 0.12 mg/ml)	Panax notoginseng	6-OHDA-induced PC12 cells	—	Up-regulating PI3K/AKT/mTOR cell proliferation pathway and AMPK/SIRT1/FOXO3 cell survival pathway	—	—	**Zhang et al., (2017a)**
(100 mg/kg)			MPTP-induced mice	Increasing Trx-1 expression, suppressing cyclooxygenase-2 over-expression and inhibiting mitochondria-mediated apoptosis			**Luo et al., (2011)**
Daidzein (50, 100 μM)	*Pueraria thomsonii* Benth. (Fabaceae)	6-OHDA-induced PC12 cells	—	Inhibiting caspase-8 and partially inhibited caspase-3 activation	—	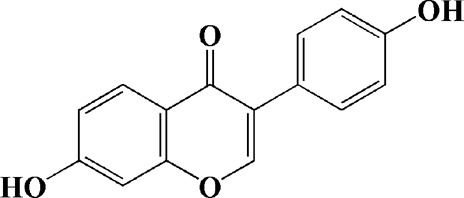	**Lin et al., (2010)**
Genistein (50, 100 μM)		6-OHDA-induced PC12 cells	—	Inhibiting caspase-8 and partially inhibited caspase-3 activation	—		**Lin et al., (2010)**
Water extract of Cyperi Rhizoma (50 and 100 mg/ml)	Cyperi Rhizoma	6-OHDA-induced PC12 cells	—	Inhibiting generation of ROS and nitric oxide, reduction of mitochondrial membrane potential, and caspase-3 activity	—	—	**Lee et al., (2010)**
Salvianic acid A (10, 50, and 100 mg/ml)	Salvia miltiorrhiza	(MPP+) -induced SH-SY5Y cells	—	Decreasing Bax/Bcl-2 ratio; inhibiting the activation of caspase-3 and cytochrome c release; protecting MMP	—	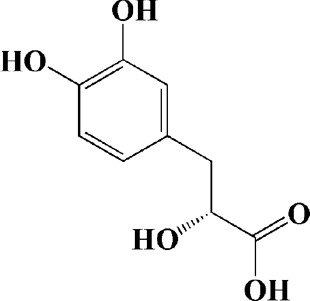	**Wang and Xu, (2005)**
Verbascoside (0.1, 1 and 10 μg/ml)	*Buddleja officinalis* Maxim	(MPP+) -induced PC12 cells	—	Increasing extracellular hydrogen peroxide level, the activation of caspase-3 and the collapse of MMP.	—	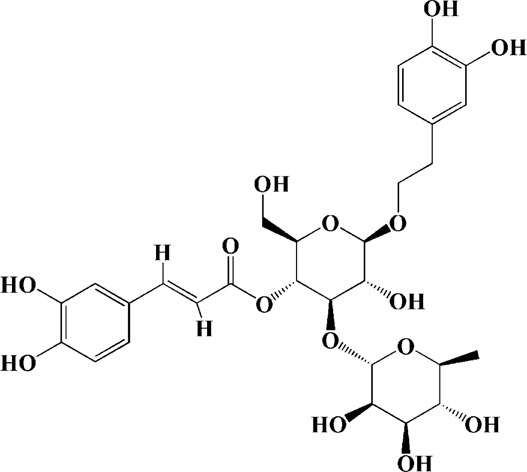	**Sheng et al., (2002)**
Notoginsenoside R2 (20 μM)	Panax ginseng	6-OHDA-induced SH-SY5Y cells	—	Activating MEK1/2-ERK1/2 pathways; activating P90RSK, inactivating BAD; inhibiting MMP depolarization		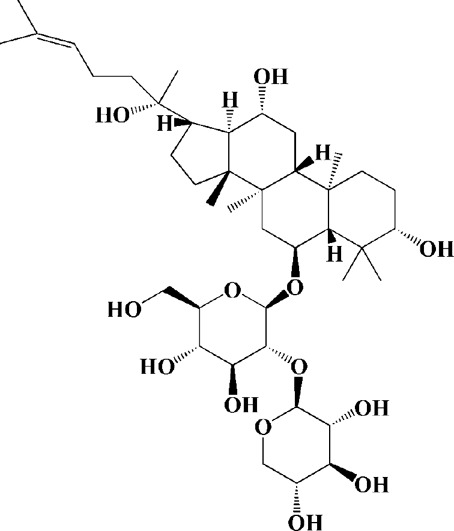	**Meng et al., (2013)**
Gingenoside Rg1 (50 μM)		Rotenone-induced substantia nigral neurons	—	Preventing cytochrome c release from the mitochrondrial membrane and increasing the phosphorylation inhibition of the pro-apoptotic protein Bad through activation of the PI3K/Akt pathway		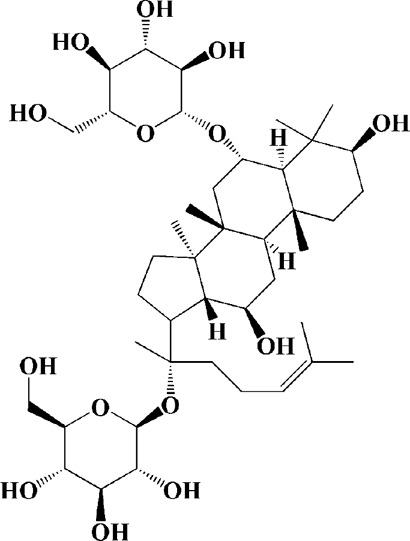	**Radad et al., (2004a)**
(10 μM)		Glutamate-induced mesencephalic dopaminergic cells	—	Increasing MMP; inhibiting apoptosis			**Leung et al., (2007)**
Ginsenosides Rb1 (10 μM)		Glutamate-induced mesencephalic dopaminergic cells	—	Increasing MMP; inhibiting apoptosis		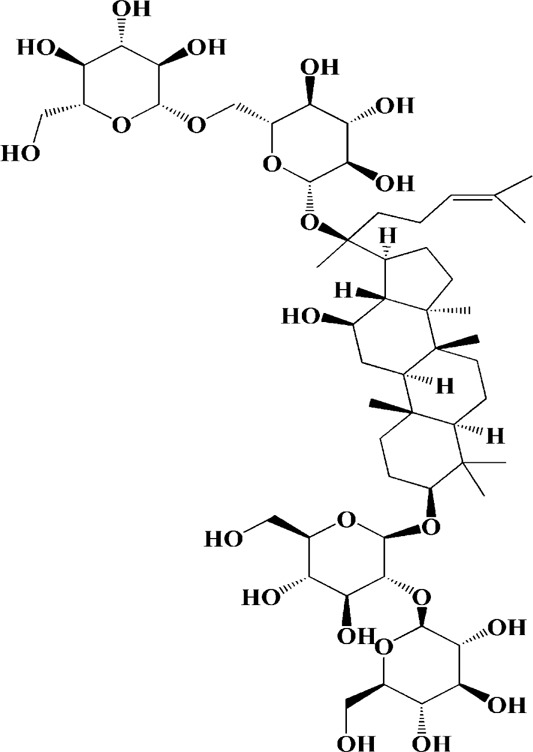	**Radad et al., (2004b)**
Water extract of Panax ginseng (0.2 mg/ml)		(MPP+) -induced SH-SY5Y cells	—	Inhibiting overproduction of ROS, elevated Bax/Bcl-2 ratio, release of cytochrome c and activation of caspase-3 expression	—	—	**Hu et al., (2011)**
Cinnamaldehyde (5, 10 μM)	*Cinnamomum verum*	6-OHDA-induced PC12 cells	—	Decreasing ROS content and cyt-c; reducing P-p44/42/p44/42 levels	—	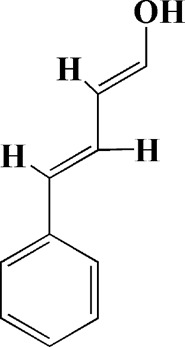	**Ramazani et al., (2020)**
Extract of *Chrysanthemum* morifolium (1, 10, 100 μg/ml)	*Chrysanthemum morifolium* Ramat	(MPP+) -induced SH-SY5Y cells	—	Attenuating the elevation of ROS level, increase in Bax/Bcl-2 ratio, cleavage of caspase-3 and PARP proteolysis	—	—	**Kim et al., (2009)**
Acacetin (2.5, 5, 10 μM)		6-OHDA-induced SH-SY5Y cells	—	Inhibiting mitochondrialmediated cascade apoptotic cell death through regulating ROS production and MMP dysfunction; reducing phosphorylation of JNK, MAPK, PI3K/Akt, and GSK-3β		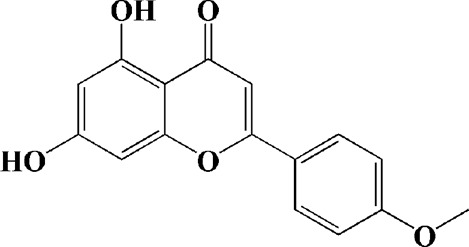	**Kim et al., (2017)**
Echinacoside (0.1, 1, 10 μM)	Cistanche salsa	6-OHDA-induced PC12 cells	—	Increasing oxidation-reduction activity and MMP; inhibiting mitochondria-mediated apoptosis		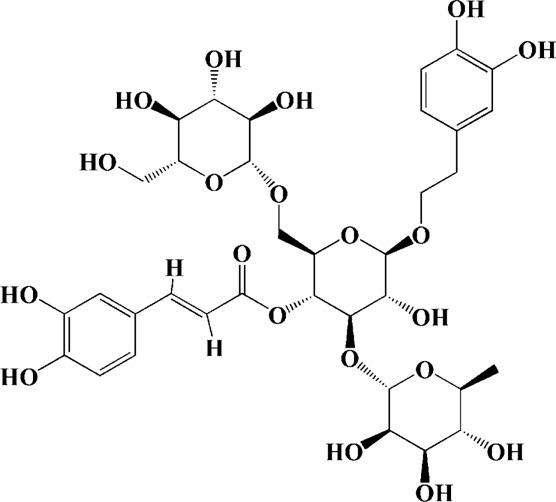	**Wang et al., (2015a)**
Ginkgetin (800 mg/kg/d)	*Ginkgo biloba* L	—	MPTP-injected mice	Decreasing the levels of intracellular ROS and maintaining MMP; inhibiting caspase-3 and Bcl2/Bax pathway		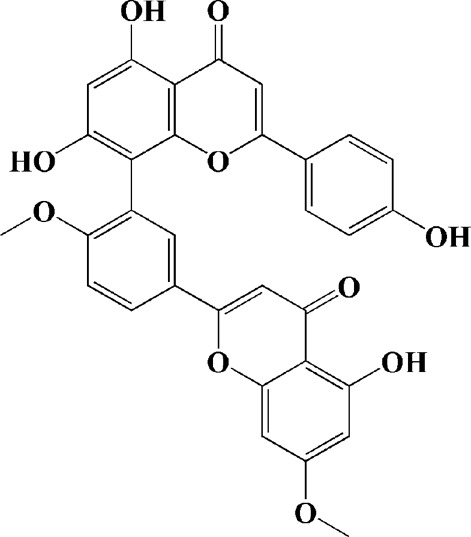	**Wang et al., (2015b)**
Gynostemma pentaphyllum polysaccharides (50 μg/ml)	Gynostemma (G.) pentaphyllum	(MPP+) -induced PC12 cells	—	Inhibiting elevated Bax/Bcl-2 ratio, as well as the release of cytosolic cytochrome c; attenuating caspase-3/9 activation and cleavage of PARP	—	—	**Deng and Yang, (2014)**
2,3,5,4 - tetrahydroxystilbene-2-O-β-D-glucoside (3.125, 6.25, 12.5, 25, 50 μM)	Polygonum multiflorum	(MPP+) -induced SH-SY5Y cells	—	Inhibiting the elevation of intracellular ROS and the disruption of MMP; suppressing the upregulation of the ratio of Bax to Bcl-2 and the activation of caspase-3		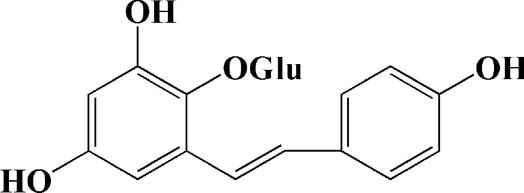	**Sun et al., (2011)**
(20 mg/kg)		—	MPTP-induced mice	Blocking the activation of JNK and P38; downregulating of the bax/bcl-2 ratio; reversing the release of cytochrome c and smac; inhibiting the activation of caspase-3, -6, and -9	—		**He et al., (2015)**
Paeoniflorin (3, 10, 30 μM)	Paeonia lactiflora Pall	6-OHDA-induced PC12 cells	—	Inhibiting ROS/PKCd/NF-κB signaling pathway	—	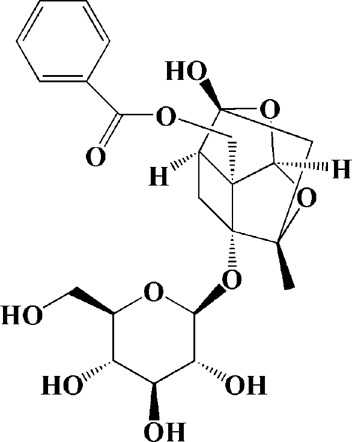	**Deng and Yang, (2014)**
Extract of Moutan Cortex Radicis (0.1, 1 μg/ml)	Moutan Cortex Radicis	(MPP+) -induced rat primary mesencephalic cells	—	Inhibiting MPTP-induced mitochondrial dysfunction; increasing expression of phosphorylated Akt, ND9, mitochondrial transcription factor A, and H2AX; inhibiting mitochondria-mediated apoptosis via the regulation of B-cell lymphoma family proteins and the inhibition of cytochrome C release and caspase-3 activation	—	—	**Kim et al., (2014)**
Tenuigenin (0.1, 1, 10 μM)	Polygala tenuifolia	6-OHDA-induced SH-SY5Y cells	—	Down-regulating caspase-3; up-regulating tyrosine hydroxylase expression		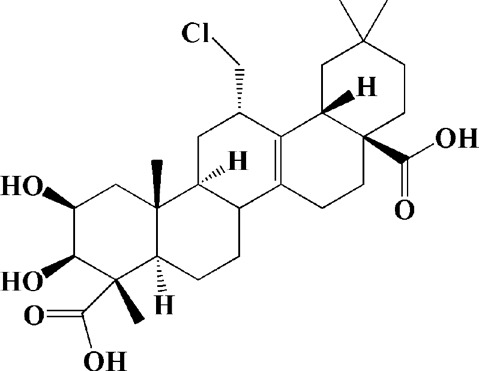	**Liang et al., (2011)**
Amentoflavone (75 μM)	Selaginella tamariscina	(MPP+) -induced SH-SY5Y cells		Inhibiting the activation of caspase-3 and p21; increasing the Bcl-2/Bax ratio; enhancing the phosphorylation of PI3K, Akt and ERK1/2		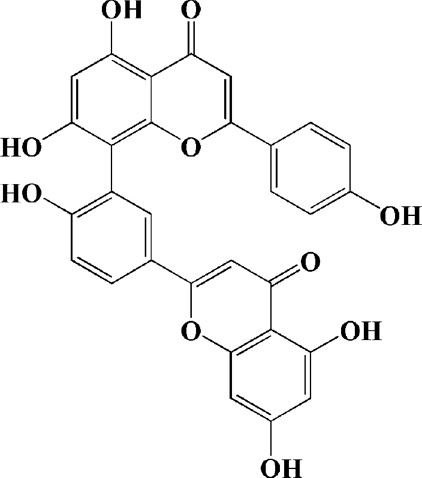	**Cao et al., (2017)**
Extract of Curcuma longa (0.05 and 0.1 mg/ml)	Curcuma longa	Salsolinol-induced SH-SY5Y cells		Reducing mitochondria-derived ROS; downregulating mRNA expression levels of p53, Bcl-2-associated X protein and caspase 3		—	**Ma and Guo, (2017)**

### 4.3 Huntington’s disease

#### 4.3.1 Cell apoptosis mediated by mitochondrial dysfunction in HD

Huntington’s chorea is an autosomal dominant neurodegenerative genetic disorder with a lower global prevalence of approximately 0.005%–0.01% than AD and PD (Bates et al., 2015). However, there are significant regional differences in the prevalence of HD. In the UK, approximately 12–13 out of 100,000 people have HD, in contrast to only 1 in every million people of Asian and African descent **(** Pringsheim et al., 2012). The clinical symptoms of HD can be manifest as chorea, dystonia and other motor disorders, as well as cognitive decline (Walker, 2007). Prior to the onset of HD, CAG trinucleotide repeat expansion in exon 1 of the Huntington (HTT) gene and leads to polyglutamine repeats in the HTT protein. Subsequently, it leads to the occurrence of mitochondrial dysfunction and preferentially leading to the loss of striatal medium spiny neurons (MSNs), and ultimately to the onset and progression of HD (Ross and Tabrizi, 2011). In HD, altered mitochondrial morphology can be considered as a hallmark event of the disease and its morphological changes are variable in different cell types. For example, in peripheral tissue cells such as lymphoblasts and fibroblasts, increased mitochondrial morphology often occurs, whereas neuronal cells exhibit increased mitochondrial fragmentation (Kim et al., 2010; Jin et al., 2013). Excessive morphological alterations will certainly lead to mitochondrial dysfunction, which in turn induces neuronal cell apoptosis. It has been shown that mitochondria in the striatum of adult rats are more sensitive to Ca^2+^-induced membrane permeability than those in the cerebral cortex and are highly susceptible to various stresses (Brustovetsky et al., 2003). In addition, the high energy demand of MSNs, the specific localization of mutant HTT (mHTT) and its aberrant interactions with brain region-specific protein partners also make MSNs more susceptible to mitochondrial dysfunction (Li et al., 2000; Goula et al., 2012).

**FIGURE 5:**
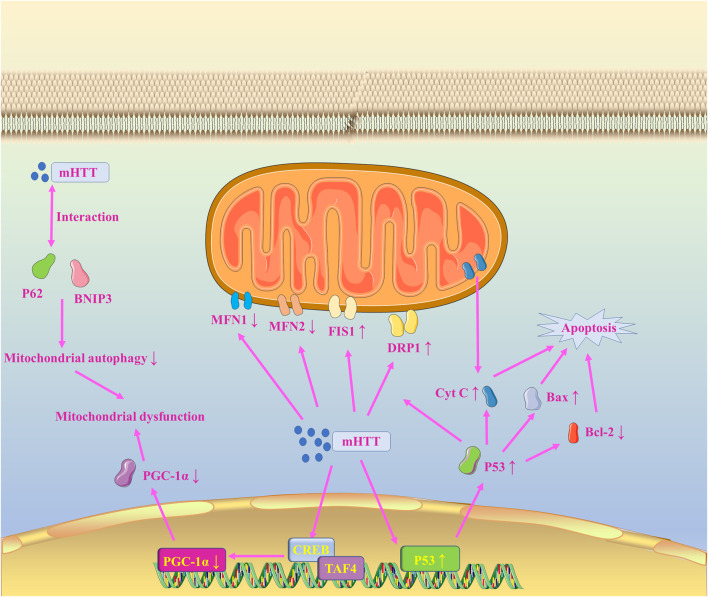
Mitochondrial dysfunction in HD.

As shown in Figure 5, HTT can affect normal mitochondrial function through multiple pathways in HD. PGC-1α, as a key transcriptional co-activator, is involved in the regulation of mtDNA production during the mitochondrial biogenesis (Puigserver and Spiegelman, 2003). However, mHTT can directly inhibit PGC-1α transcription by interfering with the CREB/TAF4 signaling pathway (Cui et al., 2006). Meanwhile, typical HD pathological features such as striatal neuron deficiency were exhibited in PGC-1α-free mice (Lucas et al., 2012). Notably, the CREB/TAF4 content did not differ significantly in striatal-like cells of wild mice and HD mice (Cui et al., 2006). This result suggests that although the interaction between mHTT and PGC-1α may result in reduced PGC-1α content, it is not the only cause of PGC-1α impairment. P53 is a tumor suppressor protein that exerts effects by regulating various target genes, including cell cycle arrest, apoptosis, and metabolism (Bae et al., 2005). In HD, mHTT binds to p53 and inhibits the recruitment of the E3 ligase Mdm2, resulting in increased p53 transcriptional activity (Bae et al., 2005). P53 has an extremely strong and sustained localization effect on mitochondria and can activate apoptosis not only by directly interacting with anti-apoptotic Bcl-2 and BAX proteins to promote BAX expression and inhibit Bcl-2 expression, but also by directly promoting the release of cytochrome C to induce apoptosis (Mahyar-Roemer et al., 2004). In previous studies, it was also found that in addition to p53 inducing mitochondrial necrosis by binding Drp1, mHTT can also promote mitochondrial fragmentation by enhancing Drp1 and FIS1 expression and inhibiting MFN1/2 expression (Shirendeb et al., 2012; Guo et al., 2013; Guo et al., 2014). Also, mHTT can interact with p62 or the autophagy protein BNIP3 to disrupt mitochondrial autophagy, further exacerbating mitochondrial dysfunction and contributing to the onset of apoptosis (Ochaba et al., 2014; Rui et al., 2015).

#### 4.3.2 Potential therapeutic agents from herbal medicine for mitochondrial dysfunction in HD

After previous studies, it has been clear that HD is closely related to mitochondrial dysfunction. However, up to now, there have been just a bit of therapeutic herbal medicine for HD, and little of herbal medicines have entered clinical studies. Among the existing research, epigallocatechin gallate and L-theanine, which are isolated from green tea, can alleviate HD symptoms of the rats induced by 3-nitro propionic acid (NP). Epigallocatechin gallate and L-theanine restored mitochondrial respiratory enzyme I, II, and IV respectively, improved REDOX enzymes such as superoxide dismutase (SOD) and catalase enzyme (CAT) **(** Kumar and Kumar, 2009a; Thangarajan et al., 2014).

As shown in Table 2, praeruptorin C is an active ingredient derived from herbal medicine *Peucedanum praeruptorum* dunn, which effectively alleviates motor deficits and depression-like behavior in HD mice induced by 3-NP. The underlying mechanism was found to be related to the upregulation of BDNF, DARPP and Huntingtin proteins in the striatum of 3-NP mice by Praeruptorin C (Wang L. et al., 2017). Puerarin, another flavonoid extracted from *Pueraria lobata*, was applied to 3-NP induced HD rat model. The results showed that puerarin could reduce apoptosis by decreasing caspase-3 activity, inhibiting mitochondrial cytochrome C release and Bax/Bcl-2 level, which was an effective method to prevent HD (Mahdy et al., 2014).

**TABLE 2:** Mitochondrial dysfunction-associated natural products for HD.

Extracts/monomers	Source	*In vitro* mode	*In vivo* model	Mechanisms or effects	Chemical structure	Refs
Epigallocatechin gallate (10, 20, 40 mg/kg/d)	Green tea	—	3-NP-induced rats	Restored mitochondrial enzyme complex (I, II, and IV) activities; attenuating MDA, nitrite concentration and restoring SOD and catalase enzyme activities	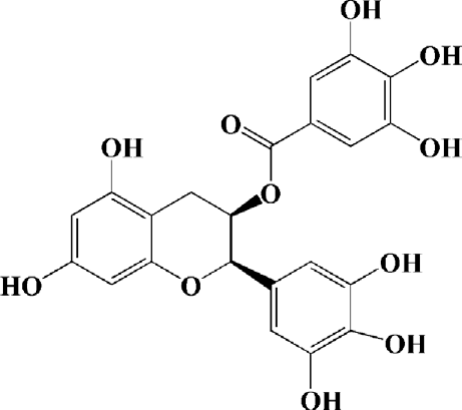	**Kumar and Kumar, (2009a)**
L-theanine (100, 200 mg/kg/d)		—	3-NP-induced rats	restored the decreased SOD, GSH, CAT, SDH and mitochondrial complexes II activity	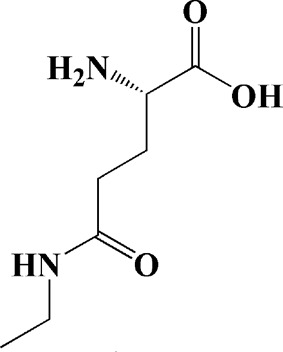	**Thangarajan et al., (2014)**
Praeruptorin C (3 mg/kg/d)	*Peucedanum praeruptorum* dunn	—	3-NP-induced mice	Upregulating BDNF, DARPP32, and huntingtin protein	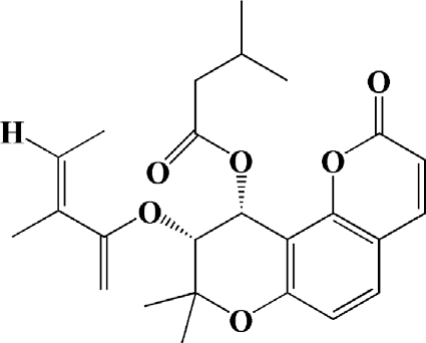	**Wang et al., (2017a)**
Puerarin (200 mg/kg/d)	Pueraria lobata	—	3-NP-induced rats	Decreasing caspase-3 activity/level, cytosolic cytochrome c, Bax/Bcl-2 levels; blocking NF-κB, TNF-α and iNOS; enhancing ATP	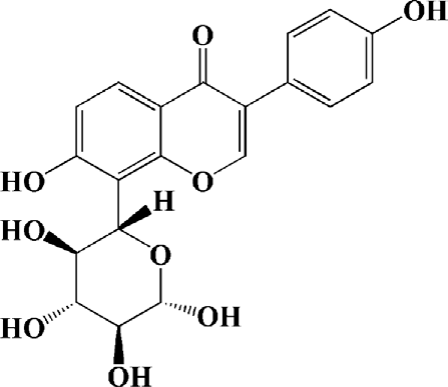	**Mahdy et al., (2014)**
Extract of Gastrodia elata (100 μg/ml)	*Gastrodia elata* Blume	PC12 cells overexpression mutant HTT genes	—	Reversing mitochondrial fragmentation and dysregulation of mitochondrial fusion and fission molecules via up-regulating the levels of MFN1, MFN2, OPA1 and down-regulating the level of FIS1	—	**Huang et al., (2019)**
(25, 50, 100 μg/ml)		PC12 cells overexpression mutant HTT genes	—	Mediating mitochondrial function and biogenesis via increasing CREB phosphorylation and expression of PGC-1α	—	**Huang et al., 2018**
Extract of *Psoralea corylifolia* Linn seed (10, 50, 100 μg/ml)	*Psoralea corylifolia* Linn	3-nitropropionic acid (3-NP)-induced	—	Reducing ATP levels, and lowering the MMP; stimulating mitochondrial respiration with uncoupling; inducing an increased bioenergetic reserve capacity	—	**Im et al., (2014)**
Aqueous extracts of *Centella asiatica* (0.01–1.0 μg/ml)	*Centella asiatica*	—	3-NP-induced mice	Enhancing the activity of succinic dehydrogenase, ETC enzymes and increasing mitochondrial viability; abolishing oxidative stress and protein oxidation in cytosol/mitochondria of brain regions	—	**Shinomol and Muralidhara, (2008)**
Extracts of *Withania somnifera* root (100, 200 mg/kg/d)	*Withania somnifera*	—	3-NP-induced rats	Promoting ATP synthesis by increasing the mitochondrial enzyme complexes (I, II, and III) in the different regions (striatum and cortex) of the brain	—	**Kumar and Kumar, (2009b)**
Lutein (50, 100 mg/kg/d)	Matrimony vine	—	3-NP-induced rats	Increasing activities of mitochondrial complexes (I, II, IV); attenuating oxidative stress	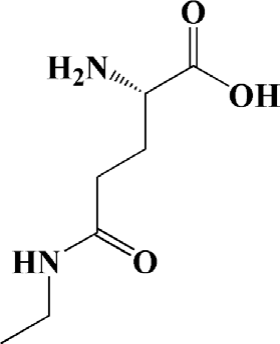	**Binawade and Hagtap, (2013)**
Celastrol (0.1, 0.5, 1 μM)	Tripterygium wilfordii	Cadmium-induced PC12 cells	—	Inhibiting activation of JNK and Akt/mTOR network; suppressing Akt/mTOR pathway by elevating PTEN (phosphatase and tensin homolog); inhibiting activation of caspase-3	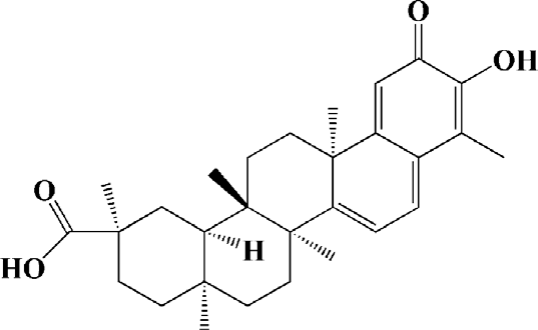	**Chen et al., (2014)**
(1 μM)		Cadmium-induced PC12, SH-SY5Y cells and primary murine neurons	—	Preventing the inactivation of AMPK by mitochondrial ROS, thus attenuating Cd-induced mTOR activation and neuronal apoptosis; inhibiting activity of caspase-3		**Zhang et al., (2017b)**
Neferine (7.5 μM)	*Nelumbo nucifera*	PC12 cells transfected with GFP-LC3	—	Reducing both the protein level and toxicity of mutant huntingtin through an autophagy-related gene 7 (Atg7)-dependent mechanism	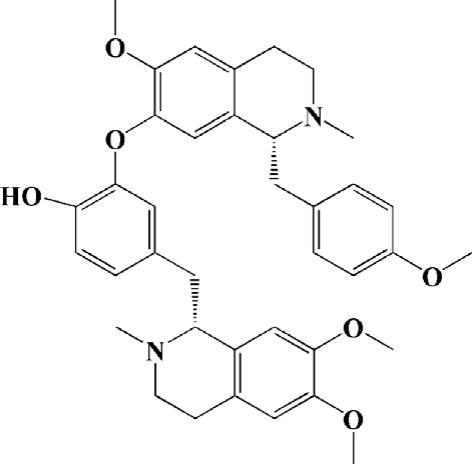	**Wong et al., (2015)**

In addition to the active monomer components, herbal extracts are also a potentially effective way to treat HD. *Gastrodia elata* is a common Chinese herbal medicine used to treat dizziness, headaches and epilepsy. In recent studies, it was found that extract of *Gastrodia elata* (EG) might have a potential therapeutic effect on HD. In PC12 cells overexpressing mutant Huntingtin protein (mTT), EG could reverse mitochondrial dynamic dysregulation by up-regulating MFN1, MFN2 and OPA1 and down-regulating FIS1. At the same time, EG could promote mitochondrial biogenesis by enhancing PGC-1 α expression and CREB phosphorylation, reducing mHTT aggregation and alleviating HD symptoms (Huang et al., 2019).

## 5 Discussion

AD, PD and HD are the main types of neurodegenerative diseases. Many studies have been carried out on the pathogenesis of AD, PD and HD in order to find effective treatment strategies. In current studies, neuroinflammation, apoptosis, autophagy and oxidative stress are all involved in the pathogenesis of neurodegenerative diseases, among which apoptosis mediated by mitochondrial dysfunction is indispensable. Mitochondria are the key centers coordinating antioxidant, energy production and apoptosis in cells. As a cell with high demand for energy, neurons have a strong dependence on mitochondria. Therefore, proper maintenance of mitochondrial function is critical for neuronal cells and is a potential therapeutic target for neurodegenerative diseases such as AD, PD and HD. Herbal medicine is widely used in modern drug development because of its multi-function and multi-target function. In this review, we focus on apoptosis-related mitochondrial dysfunction and related potential therapeutic herbs. The results show that herbal medicines can be effective against mitochondrial dysfunction in a variety of models of AD, PD and HD, and are a class of potential drug sources with outstanding effects. However, in the current study, there are still many herbal medicines that have not fully elucidated the specific relationship between them and mitochondrial dysfunction, and further research is needed. All in all, herbal medicine can be used as an important class of drugs for the treatment of neurodegenerative diseases and needs further development.
