# Supplementary material for: Regulation of mitochondrial dysfunction induced cell apoptosis is a potential therapeutic strategy for herbal medicine to treat neurodegenerative diseases
Source: Front Pharmacol. 2022 Sep 22;13:937289. doi: 10.3389/fphar.2022.937289 (PMC9535092; doi:10.3389/fphar.2022.937289)
Supplement: Supplementary file 1 [file Table1.docx]

**Supplementary Table 1** Mitochondrial dysfunction-associated natural products for AD.

| **Extracts/**  **monomers** | **Source** | ***In vitro*** | ***In vivo*** | **Mechanisms or effects** | **Clinical trial** | **Chemical structure** | **Refs.** |
| --- | --- | --- | --- | --- | --- | --- | --- |
| Ginsenoside Rb1  (12.5, 25, 50 mg/kg/d) | Panax ginseng | — | Aβ_1-40_-injected Kunming mice | Upregulating Bcl-2 expression; declining bax and cleaved-caspase-3 expressions | — |  | **(Wang et al., 2018)** |
| Ginsenoside Rg1  (20 mg/kg/d) |  | — | 3xTg-AD mice harboring PS1M146V, APPSwe, and TauP301L transgenes | Upregulating complexin-2 (CPLX2), synapsin-2 (SYN2), synaptosomal-associated protein 25 (SNP25) | — |  | **(Nie et al., 2017)** |
| Ginsenoside Rg3  (20 mg/kg/d) | Panax ginseng | — | D-gal administered Wistar rats | Upregulating Bcl-2 expression; declining caspase-3, caspase-9, Bax, AIF and Cyt C expressions; regulating disordered amino acid metabolism | — |  | **(Lee et al., 2013)** |
| Ginsenoside Rd  (10μM) | Panax ginseng | Aβ_25–35_-induced primary hippocampal neurons | ­­— | Increasing the expression of Bcl-2 mRNA; decreasing the expressions of Bax mRNA and Cyt c mRNA; downregulating the protein level of cleaved Caspase-3 | — |  | **(Liu et al., 2015)** |
| Ginsenoside Re  (20, 25, and 30 μM) | Panax ginseng | Aβ_25–35_-induced SH-SY5Y cells | — | Increasing Bcl-2/Bax ratio and Nrf2 expression; reducing cytochrome c release; inactivating caspase-3/9. | — |  | **(Liu et al., 2019)** |
| Ginsenoside Re  (1, 4mg/kg/d) | Panax ginseng | — | β-amyloid-injected mice | Restoring amino acid, lecithin, and sphingolipid metabolism. | — |  | **(Li et al., 2017)** |
| Red Ginseng Oil  (10, 50 and 100 μg/mL) | Panax ginseng | Aβ_25–35_-induced PC12 cells | — | Inhibiting calcium influx, reducing mitochondrial membrane potential loss; decreasing Bax; increasing Bcl-2 and inactivating caspase-3 and -9 and PARP-1; suppressing iNOS, COX-2, PEG2 and NO | — | — | **(Lee et al.,2017)** |
| Korean red ginseng extract  ((1mg/mL)) | Panax ginseng | H_2_O_2_-induced SK-N-SH cells | — | Decreasing expression of p-p53 and caspase-3; increasing expression of Bcl-2 and PI3K/Akt pathway | Phase II | — | **(Nguyen et al., 2015)** |
| (0.2, 2 and 20 μg/ mL) |  | Aβ-induced SH-SY5Y cells | — | Decreasing Bax/Bcl-2 ratio and caspase-3 activity; inhibiting the activation of NF-κB; enhancing MMP | — | — | **(Choi et al., 2020)** |
| (1, 10 and 100 μg/mL) |  | Aβ_1–42_-induced HT22 cell | — | Restoring ATP-linked respiration | — | — | **(Shin et al., 2020)** |
| (100 mg/kg/d) |  | — | 5XFAD mice that expressed five familial AD mutations at the human presenilin 1 (PSEN1) gene (M146 and L286) and human APP gene (K670N/M671L, V717I, and I716V) | Regulating mitochondrial fusion and fission | — | — | **(Shin et al., 2020)** |
| Puerarin  (100 μM) | Pueraria lobate | Aβ_25–35_-induced PC12 cells | — | Increasing the expression of P-Akt, Bcl-2 and p-Bad; decreasing expression of Bax and cytochrome c release | — |  | **(Xing et al., 2011)** |
| (0.1, 1 and 10 μM) |  | SAD cells | — | Decresing Bax/Bcl-2 ratio and expression of caspase-3, p38 and JNK | — |  | **(Zhang et al., 2011)** |
| Urolithin A  (10 μM) | Pomegranate fruits | SH-SY5Y cells transfected with the amyloid-precursor protein 695 | — | increase expression of genes for mitochondrial biogenesis and OXPHOS | — |  | **(Esselun et al., 2020)** |
| Honokiol  (5 and 10 μM) | Magnolia ofﬁcinalis | Aβ_1–42_-induced PC12 cells | — | Inhibiting the activation of GSK-3β; attenuating the nuclear accumulation of β-catenin; suppressing the phosphorylation of β-catenin (Ser33/Ser37/Thr41 site), the release of cytochrome c | — |  | **(Xian et al., 2016)** |
| (0.7, 7, and 70 μg/kg/d) |  | — | ICV- Aβ_1–42_- injected mice | Inhibiting NF-kB pathway; | — |  | **(Wang et al., 2017b)** |
| (10, 50 and 100 μM) |  | Aβ-oligomers-induced Primary cells of hippocampal neurons | — | Inhibiting NF-kB pathway; decreasing the expression of Bax, cleaved-caspase 9 and 3, cytochrome c; increasing expression of Bcl-2 | — |  | **(Wang et al., 2017b)** |
| 4-O-methylhonokiol  (1 mg/kg/d) | Magnolia ofﬁcinalis | — | C57BL/6 mice expressing the NSE/AβPPsw fusion gene | Decreasing expression and activity of BACE1; inactivating caspase-3 and Bax | — |  | **(Choi et al., 2011)** |
| Berberine  (0.3-10μM) | Coptidis Rhizoma | CHO-APP695-induced CHO cells | — | Increasing binding affinities to PPARγ protein to modulate the mitochondrial bioenergetics. | — |  | **(Wong et al., 2021)** |
| Pinocembrin  (1, 10 and 20 μM) | Propolis and several plants | Aβ_25–35_-induced SH-SY5Y cells | — | Increasing membrane potential and Bcl-2/Bax ratio; suppressed the release of cytochrome c and the cleaved caspase-3; activating Nrf2/HO-1 pathway | — |  | **(Wang et al., 2016b)** |
| Carnosic acid  (10 μM) | Rosmarinus officinalis L. | Aβ_42_-induced SH-SY5Y cells | — | Suppressing caspase3/8/9 expressions | — |  | **(Meng et al., 2014)** |
| Isorhynchophylline  (20, 40 mg/kg/d) | Uncaria rhynchophylla | — | Aβ_25–35_-injected mice | Down-regulating GSK-3β activity; activating PI3K/Akt signaling pathway; down-regulating the protein and mRNA levels of the ratio of Bcl-2/Bax, cleaved caspase-3 and caspase-9 | — |  | **(Xian et al., 2014)** |
| (1, 10 and 50 μM) |  | Aβ_25–35_-induced PC12 cells |  | Inhibiting GSK-3β activity; activating PI3K/Akt signaling pathway; elevating protein level of p-CREB |  |  | **(Xian et al., 2013)** |
| Salidroside  (10, 50 and 100 μM) | Rhodiola rosea | Aβ_25-35_-induced SH-SY5Y cells | — | Suppressing expression of Bax; increasing Bcl-XL; recovering MMP; inhibiting phosphorylation of JNK and p38 MAP kinase | — |  | **(Zhang et al., 2010)** |
| (50 μM) |  | Aβ_1–42_-induced PC12 cells | — | Activating ERK1/2 and AKT signaling pathways; recovering MMP; inhibiting caspase-3/7 activity | — |  | **(Liao et al., 2019)** |
| (20, 40 mg/kg/d) |  | — | D-gal-injected rats | Reducing Bax/Bcl-2 ratio and caspase-9 level | — |  | **(Gao et al., 2015)** |
| β-asarone  (7.5, 15, 30 μg/ml) | Acorus tatarinowii | Aβ_25–35_-induced PC12 cells | — | Reducing Aβ-induced JNK activation; down-regulating Bcl-w and Bcl-xL; inhibiting mitochondrial release of cytochrome c and activation of caspase-3 | — |  | **(Li et al., 2010)** |
| (12.5、25 and 50 mg/kg/d) |  | — | Aβ_1–42_-injected rats | Up-regulation of Bcl-2, Bcl-w; inhibiting caspase-3 activation and JNK phosphorylation | — |  | **(Yang et al., 2021)** |
| Paeoniﬂorin  (7.5, 15 and 30 mg/kg/d) | Radix Paeoniae Alba | — | Rats stereotaxically injected Aβ_1-42_ | Restoring Ca^2+^ homeostasis | — |  | **(Zhong et al., 2009)** |
| (2, 10, 50μM) |  | Aβ_25-35_-induced SH-SY5Y cells | — | Increasing mitochondrial membrane potential; decreasing Bax/Bcl-2 ratio, CytC release and caspase 3/9 activities | — |  | **(Wang et al., 2014b)** |
| L-3-n-Butylphthalide  (10 and 30 mg/kg/d) | Umbelliferous  plants | — | Aβ_1–40_-injected rats | Blocking caspase-3 activation; reducing activation of GSK-3β and tau protein phosphorylation | Phase II |  | **(Peng et al., 2009)** |
| (0.1, 1, 10μM) |  | Aβ_25-35_-induced SH-SY5Y cells | — | Decreasing caspase-3, caspase-9, and cytochrome c expressions; increasing Bcl-2 expressions; increasing MMP; activating MAPK pathway |  |  | **(Lei et al., 2014)** |
| Asiatic acid  (0.01 to 1.0 μM) | Centella asiatica | C2-ceramides-induced primary cultured rat cortical neuronal cells | — | Reducing the cytosolic release of HtrA2/Omi; down-regulating Bax, caspase 3, and the dephosphorlyation of ERK1/2; increasing MMP | — |  | **(Zhang et al., 2012)** |
| (5, 10 and 20 μM) |  | Aβ_25–35_-induced PC12 cells | — | Preventing IκBα degradation and p65 nuclear translocation; promoting the phosphorylation of Akt and GSK-3β | — |  | **(Cheng et al., 2018)** |
| (75 mg/kg/d) |  | — | AlCl^3^-induced Wistar rats | Activating Akt/GSK3β pathway; decreasing caspases-3, -6, -8, -9, Bax and cytochrome c (cytosol) expression; increasing Bcl-2 and cyto-c (mitochondria) | — |  | **(Ahmad Rather et al., 2019)** |
| Asiaticoside | Centella asiatica | — | Aβ_1–42_-injected rats | Decreasing caspase 3 expression; increasing Bcl-2 expression | — |  | **(Zhang et al., 2016c)** |
| Tenuifolin  (20, 40 and 80 mg/kg/d) | Polygala tenuifolia | — | APP/PS1 transgenic AD mice | Protecting mitochondrial structure and neurons | — |  | **(Wang et al., 2019)** |
| (50μM) |  | Aβ_25-35_-induced SH-SY5Y cells | — | Increasing MMP; inhibiting the activation of caspases-3 and -9 | — |  | **(Wang et al., 2019b)** |
| Shikonin  (3.47, 10.42, 34.72 µM) | Lithospermum erythrorhizon | Aβ_1–42_-induced PC12 cells | — | Reducing the activity of caspase-3 and the ratio of Bax/Bcl-2; increasing MMP | — |  | **(Tong et al., 2018)** |
| Hydroxytyrosol  (5 mg/kg/d) | Olive cultivars | — | AβPP/PS1 transgenic mice | Reduced mitochondrial carbonyl protein; upregulating HO-1 and NQO-1 expression; enhancing SOD-2 expression; restoring mitochondrial (II, III, and IV) activities; restoring JNK/MAPK signaling | — |  | **(Peng et al., 2016)** |
| Epigallocatechin-3-gallate  (1μM) | Camellia sinensis | N2a cells transfected with AβPP_sw_ | — | Increasing MMP; restoring respiratory rates | Phase II |  | **(Dragicevic et al., 2011)** |
| (37.1 mg/kg/d) |  | — | AβPP/PS-1 mice | Increasing MMP; restoring respiratory rates |  |  | **(Dragicevic et al., 2011)** |
| (10 μM) |  | N2a cells stably transfected with the human APP695 | — | Suppressing the transcription and translation of BACE1; promoting PPARγ mRNA and protein expressions; decreasing the expression of Bax, caspase-3 |  |  | **(Zhang et al., 2017d)** |
| Evodiamine  (5, 20 μM) | Evodia rutaecarpa | L-glutamate (L-Glu)-induced HT22 cells | — | Increasing the B-cell lymphoma-2 protein content, and inhibited the high expression levels of Bax, Bad, and cleaved-caspase-3 and -8 | — |  | **(Zhang et al., 2018)** |
| Huperzine A  (1 μM) | Huperzia serrata | Aβ_25–35_-induced PC12 cells | — | Increasing MMP; protecting mitochondrial morphology by increasing the activities of Na^+^/K^+^-ATPase, complex II/III and Complex IV | Phase II |  | **(Gao et al., 2006)** |
| (0.1, 1, 10 μM) |  | Aβ_42_-induced cortical neurons | — | Increasing ATP and MMP; inhibiting ROS overproduction |  |  | **(Lei et al., 2015)** |
| (0.01, 0.1, 1 μM) |  | — | Aβ_42_-induced AβPP/PS1 mice | Increasing the enzymatic activities of complex II-III and complex IV |  |  | **(Yang et al., 2012)** |
| (0.1 mg/kg/d) |  | — | APPswe/PS1dE9 mice | Reducing the deposition of Aβ and the ABAD level; decreasing the release of cytochrome-c and the level of cleaved caspase-3 |  |  | **(Xiao et al., 2019)** |
| Schisanhenol  (10, 30, 100 mg/kg/d) | Schisandra chinensis | — | Scopolamine-induced mice | Increasing the levels of SIRT1 and PGC-1α; decreasing the phosphorylated Tau protein (Ser 396) | — |  | **(Han et al., 2019)** |
| Quercetin  (10, 20, 40, and 80 μM) | Several plants | Aβ_25–35_-induced PC12 cells | — | Decreasing HO-1 protein; increasing sirtuin1, and Nrf2 protein | — |  | **(Yu et al., 2020)** |
| (20, 40 mg/kg/d) |  | — | APPswe/PS1dE9 transgenic mice | Increasing AMPK activity and MMP | — |  | **(Wang et al., 2014a)** |
| Isoquerctrin  (10, 20 μM) | Several plants | Aβ-induced MC65 cells | — | Decreasing caspase-3, -9 activity and increasing MMP | — |  | **(Carmona et al., 2020)** |
| (5 μM) |  | Streptozotocin-induced N2a cells | — | Up-regulating expression of Bcl-2; down-regulating expression of Bax and cleaved caspase-3; enhancing expression of VDAC; |  |  | **(Chen et al., 2020)** |
| Silibinin  (100, 200 mg/kg/d) | Silybum marianum | — | APP/PS1 transgenic mice | Suppressing the activation of caspase-3 by inhibiting Jun N-terminal kinase phosphorylation and the downstream hippocampal Bax/Bcl-2 ratio; | — |  | **(Bai et al., 2017)** |
| Nobiletin  (3.125-25 μM) | Citrus species | H_2_O_2_-induced PC12 cells | — | Decreasing caspase-3 activity and increasing MMP | — |  | **(Lu et al., 2010)** |
| (10 and 30 μM) |  | Aβ_1–42_-induced hippocampus neuron cells | — | Increasing CREB phosphorylation | — |  | **(Matsuzaki et al., 2006)** |
| Tanshinone IIA  (0.1, 1 and 10 μM) | Salvia miltiorrhiza | Aβ_25–35_-induced primary cortical neurons cells | — | Reducing caspase-3 activity, and cytochrome c translocation; increasing Bcl-2/Bax ratio and MMP | — |  | **(Liu et al., 2010)** |
| Andrographolide  (20 μM) | Andrographis paniculate | Aβ_1–42_-induced PC12 cells | — | Activating Nrf2/p62 pathway; activating expression of Beclin-1 and LC3; increasing MMP; reducing the levels of ROS, Cytc, and Bax | — |  | **(Gu et al., 2018)** |
| Lycopene  (2.5 and 5mg/kg/d) | Many fruits | — | Aβ_1–42_-induced rats | Inhibiting oxidative, mitochondria damage, TNF-α, IL-6 and Caspase-3 activity. | — |  | **(Prakash et al., 2014)** |
| Safranal  (0.025, 0.1, and 0.2 ml/kg/d) | Crocus sativus | — | Aβ_1–40_-induced rats | Attenuating hippocampal level of ROS, protein carbonyl, IL-1β, IL-6, TNF-α, NF-kB, caspase 3 and DNA fragmentation; increasing MMP. | — |  | **(Baluchnejadmojarad et al., 2019)** |
| Crocin  (0.5 and 2 μM) | Crocus sativus | L‑glutamate‑damaged HT22 cells | — | Reducing the apoptotic rate, mitigated mitochondrial dysfunction; suppressing ROS accumulation and Ca^2+^ overload; decreasing the expression levels of Bax, Bad and cleaved caspase‑3 and increasing the expression levels of B‑cell lymphoma‑extra large, phosphorylated (P‑) protein kinase B and P‑mammalian target of rapamycin | — |  | **(Wang et al., 2019a)** |
| Linalool  (100 μM) | Several aromatic plants | Glutamate-induced HT-22 cells | — | Reducing cell apoptosis; reducing mitochondrial ROS and mitochondrial calcium levels; preserving MMP | — |  | **(Sabogal-Guáqueta et al., 2019)** |
| (100 mg/kg/d) |  | — | Aβ_1–40_-injected mice | Suppressing cleaved caspase (caspase-3, caspase-9); elevating Nrf2 and HO-1 expression | — |  | **(Xu et al., 2017b)** |
| Xanthoceraside  (0.5 and 2 μM) | Xanthoceras sorbifolia | Aβ_25–35_-induced SH-SY5Y cells | — | Decreasing apoptosis, ROS overproduction, MMP dissipation, intracellular calcium overload, and caspase-3 activity | — |  | **(Chi et al., 2013)** |
| 6‴-Feruloylspinosin | Ziziphus jujuba var. spinosa | Aβ_1–42_-induced PC12 cells | — | Promoting the expression of Pink1/Parkin | — |  | **(Yang et al., 2020)** |
| Ginkgo biloba extracts  (20mg/kg/d) | G. biloba | — | Aβ_25–35_-injected rats | Inhibiting Bax expression, cytochrome c release, and the activity of caspase-9/3. | Phase II | — | **(Tian et al., 2013)** |
| Hydroxy-α-sanshool  (15, 30 and 60 μM) | Zanthoxylum bungeanum | H_2_O_2_-stimulated  PC12 cells | — | Upregulating the expressions of p-PI3k, Akt, p-Akt, and Bcl-2, downregulating the expressions of cleaved caspase-3 and Bax | — |  | **(Li et al., 2020; Li et al., 2022)** |
| Ethanolic fraction of the seeds  (0.1 – 10 μg/mL) | Cassiae Semen | NMDA-induced mouse primary hippocampal cells | — | Attenuating secondary Ca^2+^ dysregulation | — | — | **(Drever et al., 2008)** |
| Ampelopsin  (100 and 200 mg/kg/d) | Ampelopsis grossedentata | — | D-gal-induced aging rat | Decreasing the expression of caspase-3, p53 and p62; up-regulating Bcl-2 and SIRT1 activity; suppressing aging-related astrocyte activation and inhibiting mTOR signal pathway as well as down-regulating miR-34a | — |  | **(Kou et al., 2016)** |
